# Single-cell RNA sequencing highlights the role of epithelial-immune dual features of proximal tubule cells in BK polyomavirus nephropathy

**DOI:** 10.1128/jvi.01394-25

**Published:** 2025-10-07

**Authors:** Feng Yang, Xutao Chen, Hui Zhang, Shicong Yang, Huifei Yang, Peisong Chen, Guodong Zhao, Yingzhen He, Siyan Meng, Dongfeng Yin, Qian Li, Jiang Qiu, Gang Huang

**Affiliations:** 1 Organ Transplant Center, The First Affiliated Hospital of Sun Yat-sen University 71068 , Guangzhou, China; 2 Department of Pharmacy, General Hospital of Xinjiang Military Command, Urumqi, China; 3 Department of Urology, Sun Yat-sen Memorial Hospital, Sun Yat-Sen University 26469 , Guangzhou, China; 4 Department of Pathology, The First Affiliated Hospital of Sun Yat-sen University 71068 , Guangzhou, China; 5 Department of Pathology, Fuda Cancer Hospital 425699, Guangzhou, China; 6 Department of Laboratory Medicine, The First Affiliated Hospital of Sun Yat-sen University 71068 , Guangzhou, China; 7 Guangdong Provincial Key Laboratory of Organ Donation and Transplant Immunology, Guangzhou, China; 8 Guangdong Provincial International Cooperation Base of Science and Technology (Organ Transplantation), Guangzhou, China; College of Agriculture & Life Sciences, University of Arizona, Tucson, Arizona, USA

**Keywords:** BKPyVN, scRNA-seq, microenvironment, proximal tubule cells, T-cell exhaustion

## Abstract

Up to 10% of renal allograft failures are caused by BK polyomavirus nephropathy (BKPyVN). However, there is no specific antiviral agent for BKPyVN. The only treatment is to reduce the levels of immunosuppression, which is not always practical and increases the risk of rejection. Since targeting the microenvironment is a promising approach, we performed single-cell RNA sequencing (scRNA-seq) on BKPyVN samples and stable allografts to obtain BKPyVN microenvironmental atlases. Interestingly, we identified a novel subpopulation of proximal tubule cells (annotated as IGKC+ PT) with epithelial-immune dual features that may contribute to the progression of BKPyVN through T-cell exhaustion. Additionally, we determined that the IGKC+ PT subpopulation might serve as a non-invasive diagnostic marker through scRNA-seq of urine samples and co-immunofluorescence staining. These results improve our understanding of the BKPyVN microenvironment and may guide the development of new therapeutic and diagnostic approaches for a wide range of patients.

**IMPORTANCE:**

BKPyVN severely threatens kidney transplant recipients. Due to the lack of effective drugs against BK polyomavirus (BKPyV), reducing immunosuppressant therapy is the only treatment. Unfortunately, this approach is not always effective and increases the acute rejection risk. A growing body of research suggests that potential therapeutic targets may be identified by studying the disease microenvironment. However, traditional methods have not explained why the large number of infiltrating T cells in the BKPyVN microenvironment does not effectively clear BKPyV. Newly available large-scale scRNA-seq technology can be used to study gene expression at a single-cell resolution, offering a new way to investigate the BKPyVN microenvironment. By combining scRNA-seq with experimental analysis, we found a novel subpopulation of proximal tubule cells (annotated as IGKC+ PT) with epithelial-immune dual features that may contribute to the progression of BKPyVN through T-cell exhaustion.

## INTRODUCTION

The prevalence of polyomavirus infection within 1–2 years after renal transplantation is as high as 50% (1, 2). Clinically, BK polyomavirus nephropathy (BKPyVN) is the most common complication, with an incidence of up to 10%. Due to the lack of effective drugs against BK polyomavirus (BKPyV), reducing immunosuppressant therapy is the only treatment. Unfortunately, this approach is not always effective and increases the acute rejection risk by 5%–25% (3, 4). Therefore, there is an urgent need to screen for novel drug targets to help develop new therapeutic and diagnostic options for BKPyVN.

A growing body of research suggests that the disease microenvironment can be targeted as a therapeutic strategy (5, 6). Previous studies observed that a large number of T cells infiltrate the BKPyVN microenvironment, but these T cells are ineffective in clearing BKPyV (7, 8). A recent study demonstrated that BKPyV overlapping peptide pools can induce PD-1 expression in T cells, a well-established biomarker of T-cell exhaustion (9). Another study documented a significant correlation between T-cell exhaustion and BKPyV clearing time in renal transplant patients (8). In the BKPyVN microenvironment, T cells may be in an exhausted state, resulting in the failure to eliminate virus-infected cells. Furthermore, proximal tubule (PT) cells serve as the natural target cells of BKPyV (10–12), and they may inhibit T-cell activation via PD1/PD-L1 under inflammatory conditions (13). Therefore, a comprehensive depiction of the ecosystem and immune phenotypes in BKPyVN at a single-cell resolution is urgently needed.

Single-cell RNA sequencing (scRNA-seq) is an unbiased method of studying the transcriptional profiles associated with kidney disease (14, 15). In this study, we used scRNA-seq data from kidney and urine samples of BKPyVN in combination with experiments to reveal the single-cell atlases of the BKPyVN microenvironment. Our findings may help facilitate the development of novel therapeutic and diagnostic modalities.

## RESULTS

### Identifying cell types in BKPyVN samples via scRNA-seq

**Fig 1:**
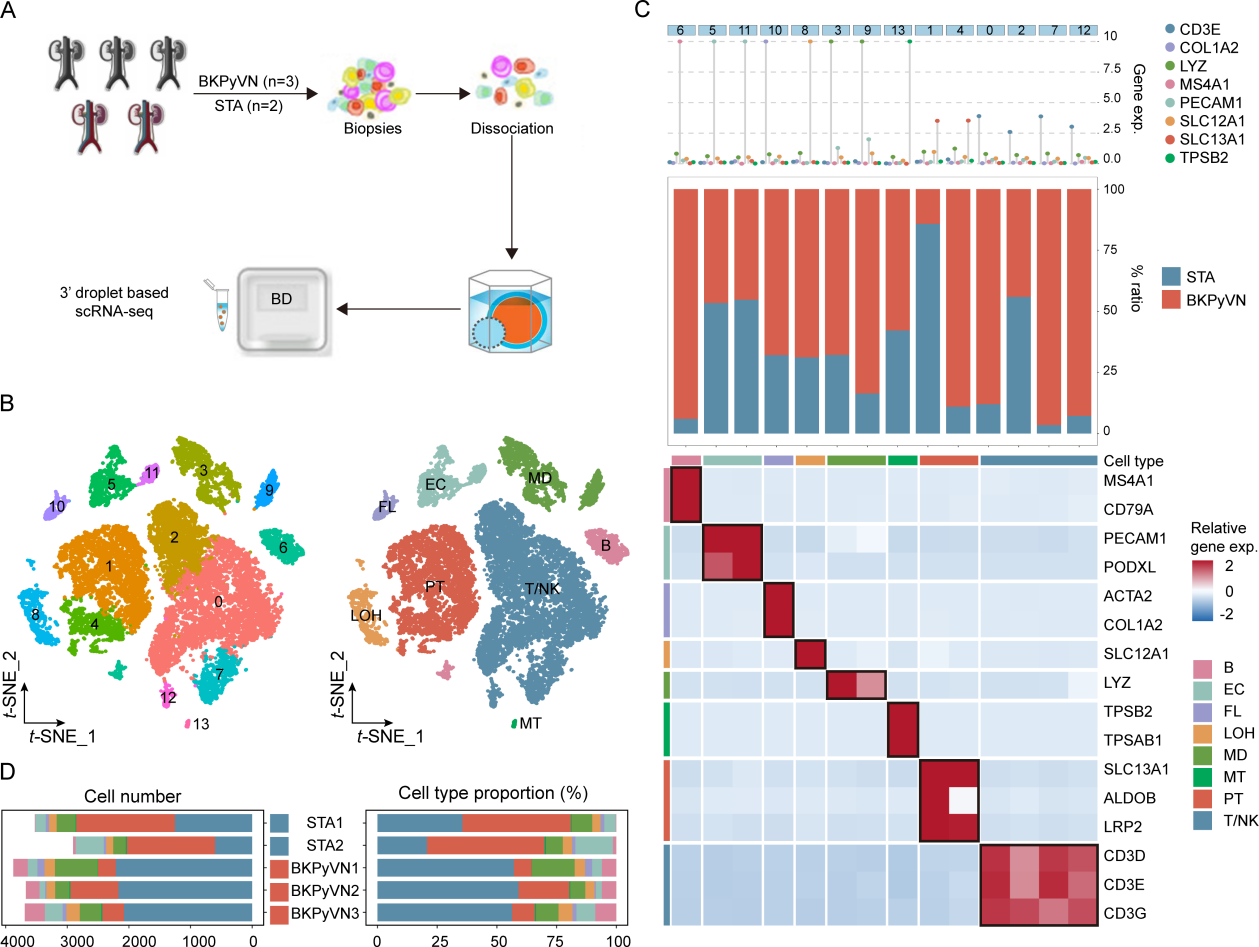
Identification of cell types in BKPyVN samples. (**A**) Summary of sample collection and scRNA-seq analysis. (**B**) Three-layered complex heatmap displaying the selected cell marker genes per cluster. The top section displays the mean expression of identified lineage markers; the middle section shows the preference of each cluster; and the bottom section presents the relative expression map of known marker genes associated with each cell subpopulation. Relative expression values are scaled by mean centering and transformed to a scale ranging from −2 to 2. (**C**) t-distributed stochastic neighbor embedding (t-SNE) plot displaying 12,856 cells from three BKPyVN and two stable allograft samples, color-coded by cell clusters or major cell types. (**D**) Bar graph presenting the number and relative proportion of major cell types in different groups.

We performed scRNA-seq on renal allograft samples from three patients with BKPyVN and two stable allograft (STA) samples (Fig. 1A). After quality control and doublet removal (Fig. S1 and S2), 12,856 single cells were clustered into 14 cell clusters (Fig. 1B). With marker-based annotations, eight major cell types were identified: PT cells (SLC13A1+, ALDOB+, and LRP2+), loop of Henle cells (SLC12A1+), fibroblast (FL) cells (ACTA2+ and COL1A1+), endothelial cells (EC) (PECAM1+ and PODXL+), T and natural killer (T/NK) cells (CD3D+, CD3E+, and CD3G+), B cells (MS4A1 + and CD79A+), myeloid (MD) cells (LYZ+), and mast (MT) cells (TPSB2+ and TPSAB1+) (Fig. 1C). Among these cells, PT and T cells were the two most abundant cell types in the disease microenvironment. Further comparison of the cellular composition in different groups revealed that the number and proportion of PT cells were significantly lower in the BKPyVN group than in the STA group, whereas the number and proportion of T/NK cells were dramatically higher. The number and proportion of other immune cells (B, MD, and MT) were also higher in the BKPyVN group (Fig. 1D).

To provide additional evidence of differences in the proportions of various cell types between the BKPyVN and STA groups, we calculated the ratio of cell types in bulk RNA-seq data from 137 samples (28 BKPyVN and 109 STA samples) using MuSiC (v ..1.1). The proportions of major immune cell types (T/NK, MD, MT, and B) were significantly higher in the BKPyVN group than in the STA group (*P* < 0.05), whereas the proportion of PT cells was significantly lower (*P* < 0.05) (Fig. S3). Together, these results suggest that PT and T/NK cells might play an essential role in the progression of BKPyVN.

### Epithelial-immune dual features of PT cells in BKPyVN samples

**Fig 2:**
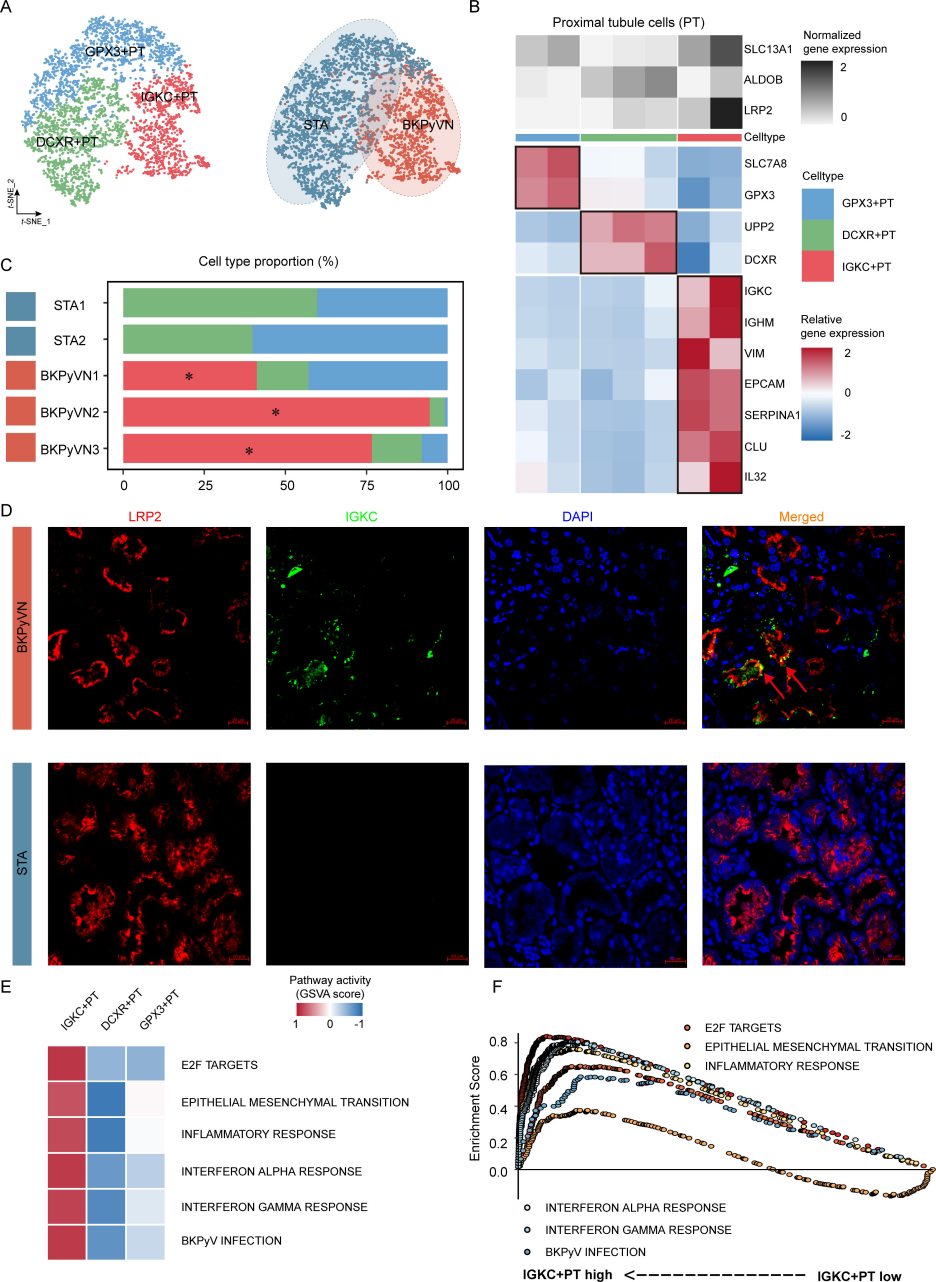
Epithelial-immune dual features of PT cells in BKPyVN samples. (**A**) Subpopulation analysis of PT cells in BKPyVN samples revealed a subpopulation (IGKC+ PT) exhibiting specific dual epithelial-immune characteristics. (**B**) Two-layered complex heatmap presenting the canonical markers for each subpopulation. Top: mean expression of PT canonical markers. Bottom: relative expression map of known marker genes associated with each cell subpopulation. Relative expression values are scaled by mean centering and transformed to a scale ranging from −2 to 2. (**C**) Relative proportion of subpopulations in different groups. (**D**) Immunohistochemical staining confirmed the presence of the IGKC+ PT subpopulation in BKPyVN samples (*n* = 6, scale bar = 20 µm). (**E**) Gene set variation analysis revealed the relationship between three PT subpopulations and BKPyVN progression. (**F**) Gene set enrichment analysis of BKPyVN samples with a high or low proportion of the IGKC+ PT subpopulation. DAPI, 4′,6-diamidino-2-phenylindole.

We performed a subpopulation analysis of PT cells to further investigate their role in BKPyVN. To confirm the absence of other cell types, PT cells were defined by the expression of SLC13A1, ALDOB, and LRP2. In total, 4,453 PT cells were clustered into three distinct cell subpopulations (Fig. 2A). We annotated these subpopulations based on the expression of canonical markers: GPX3+ PT (GPX3+ and SLC7A8+), DCXR+ PT (DCXR+ and UPP2+), and IGKC+ PT (IGKC+, IGKM+, VIM+, EPCAM+, SERPINA1+, CLU+, and IL32+) cells (Fig. 2B). The IGKC+ PT subpopulation was only found in the BKPyVN group, and its proportion tended to increase with the progression of BKPyVN (Fig. 2C). LRP2 and IGKC were co-expressed in the same cells (Fig. S4), and the IGKC+ PT subpopulation was present only in BKPyVN samples, according to the results of co-immunofluorescence staining with anti-LRP2 and anti-IGKC (Fig. 2D).

To determine the role of the IGKC+ PT subpopulation in BKPyVN, we performed gene set variation analysis (GSVA) based on BKPyVN-related signaling pathways. According to the 2019 guidelines for BKPyVN published by the American Society of Transplantation (1), the progression of BKPyVN primarily relies on evaluation of inflammation, fibrosis, and BKPyV infection. Therefore, we obtained gene sets related to inflammation, fibrosis, and BKPyV infection from hallmark gene sets and a previous study (16) (Tables S1 and S2). Compared with the DCXR+ PT and GPX3+ PT subpopulations, signaling pathways associated with BKPyVN progression were significantly enriched in the IGKC+ PT subpopulation, indicating that the IGKC+ PT subpopulation was associated with BKPyVN progression (Fig. 2E). To further illustrate the correlation between the proportion of IGKC+ PT cells and BKPyVN progression, we calculated the cell fraction of the IGKC+ PT subpopulation in the bulk RNA-seq data of BKPyVN from 28 samples using MuSiC (v.0.1.1). Based on the mean proportion of IGKC+ PT cells, we categorized BKPyVN patients into high- and low-IGKC+ PT groups. Gene set enrichment analysis (GSEA) results showed that BKPyVN progression-related signaling pathways were significantly enriched in the high-IGKC+ PT group (Fig. 2F; Tables S3 and S4). Using SCENIC (Supplemental Material S1), we also found that the transcription factor RELB, a component of NF-κB that has been reported to regulate BKPyV gene expression and replication (17, 18), acted as a specific regulator in the IGKC+ PT cells (Fig. S5). Taken together, these results indicate that the epithelial-immune dual features of IGKC+ PT cells may contribute to the progression of BKPyVN.

### Signaling pathways in the IGKC+ PT subpopulation involved in BKPyVN progression

**Fig 3:**
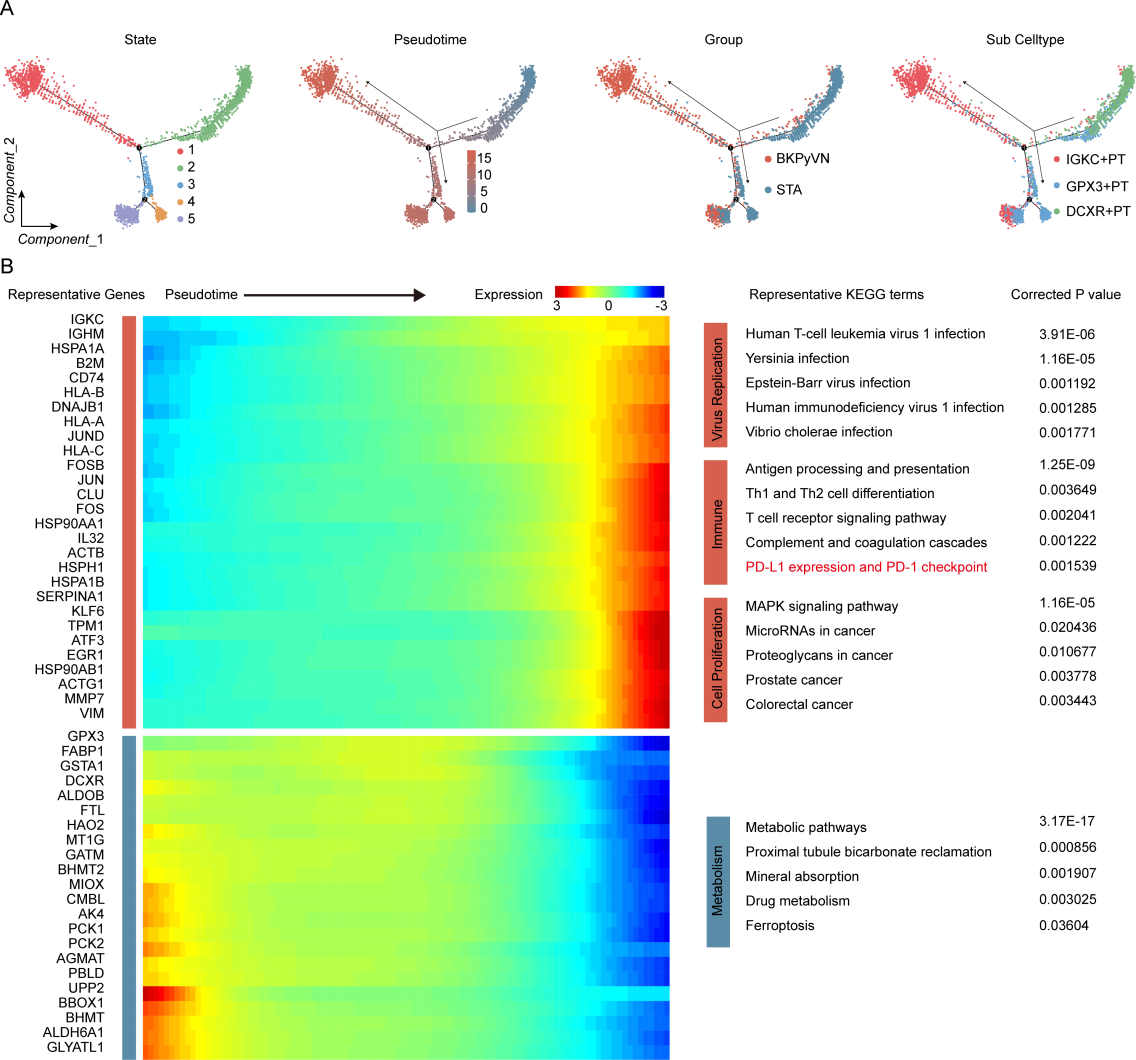
Pseudotime trajectory analysis identifying the signaling pathways of the IGKC+ PT subpopulation promoting BKPyVN progression. (**A**) Pseudotime-ordered analysis of PT cells. States, pseudotime, group, and PT subpopulation were labeled by colors. (**B**) Heatmap showing the dynamic changes in gene expression along the pseudotime axis (cataloged hierarchically into two gene modules). Corrected *P* < 0.05 was considered indicative of statistically significant KEGG pathway enrichment.

Based on pseudotime analysis, the direction of PT evolution was consistent with observed changes in groups and subpopulations (Fig. 3A). Therefore, along the pseudotime axis, significantly upregulated genes were involved in promoting the progression of BKPyVN through the IGKC+ PT subpopulation, while downregulated genes were associated with PT function in the normal state (Fig. 3B). Based on the Kyoto Encyclopedia of Genes and Genomes (KEGG) pathway enrichment analysis, the upregulated genes were enriched in viral replication, immune response, and cell proliferation, while the downregulated genes were enriched in metabolism (Fig. 3B right). Interestingly, the upregulated genes exhibited enrichment in the PD-L1 expression and PD-1 checkpoint pathway, indicating that the IGKC+ PT cells might promote T-cell exhaustion in patients with BKPyVN.

**Fig 4:**
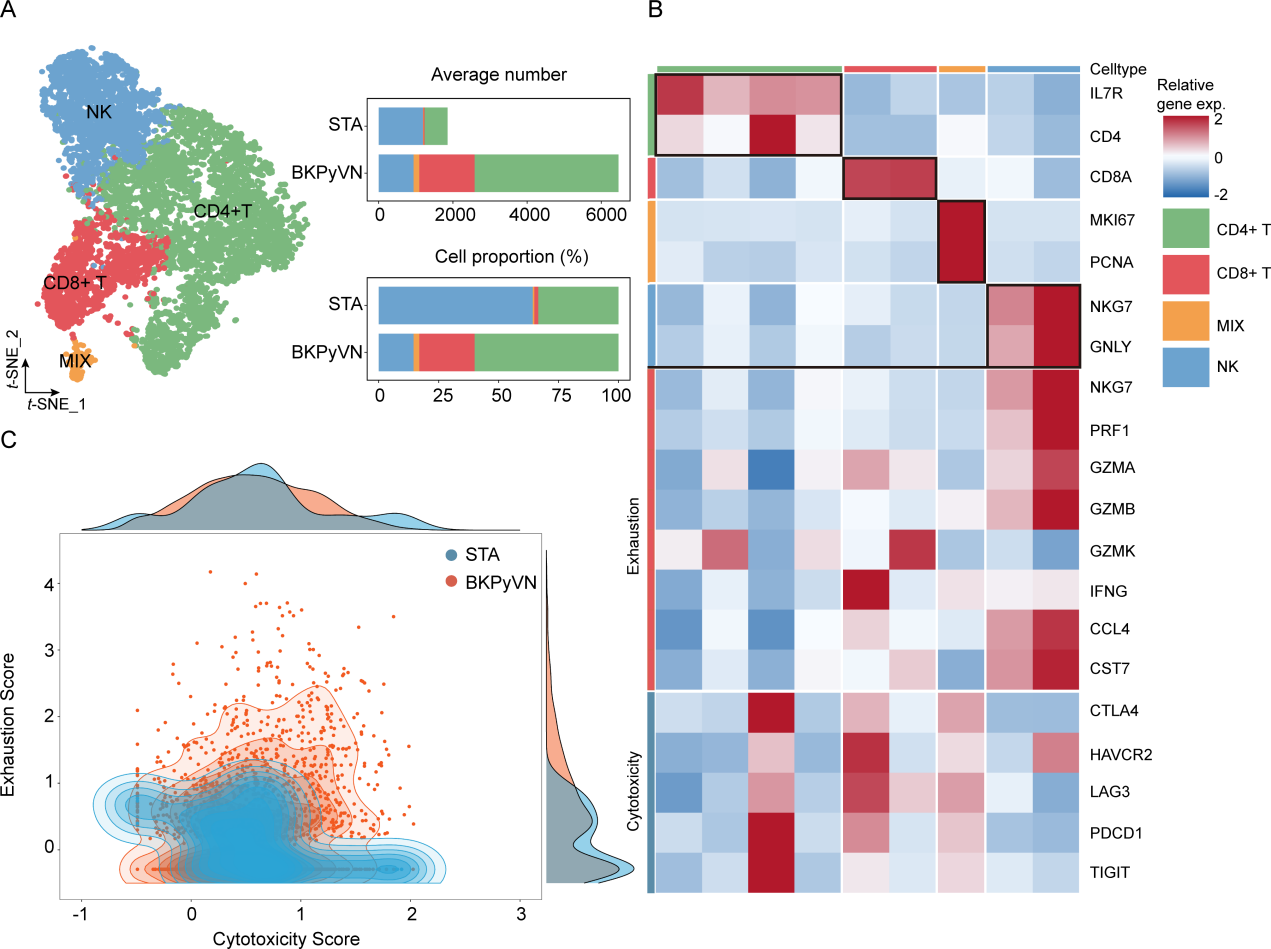
Assessing the functional states of T cells in BKPyVN samples. (**A**) t-SNE plot showing four T-cell subclusters of 8,320 T/NK cells. (**B**) Heatmap displaying the selected cell markers in each cell subset. Mean expression values are centered and scaled from −2 to 2. (**C**) Two-dimensional density plot illustrating T-cell cytotoxicity and exhaustion states in the BKPyVN and STA groups, with different colors indicating different groups.

Although prior clinical observations and our findings showed a significant increase in the number of T cells in patients with BKPyVN, it remains unclear why these T cells cannot effectively clear BKPyV. Therefore, we investigated the status of T cells in BKPyVN samples by performing a subpopulation analysis of 8,320 T/NK cells. Four distinct cell subpopulations (Fig. 4A) were identified: NK cells (NKG7+ and GNLY+), CD+ T cells (IL7R+ and CD4+), CD8+ T cells (CD8+), and MIX cells (MKI67+ and PCNA+) (Fig. 4B). In CD4+ and CD8+ T cells, we detected expression of markers representing cytotoxicity and exhaustion (Fig. 4B). We further assessed T-cell status by scoring cytotoxicity and cellular depletion and found that T cells in the BKPyVN group had lower cytotoxicity scores and higher cellular depletion scores than those in the STA group (Fig. 4C), suggesting that T cells in the BKPyVN group were in a condition of cellular exhaustion.

### Experimental validation of T-cell exhaustion in BKPyVN samples

**Fig 5:**
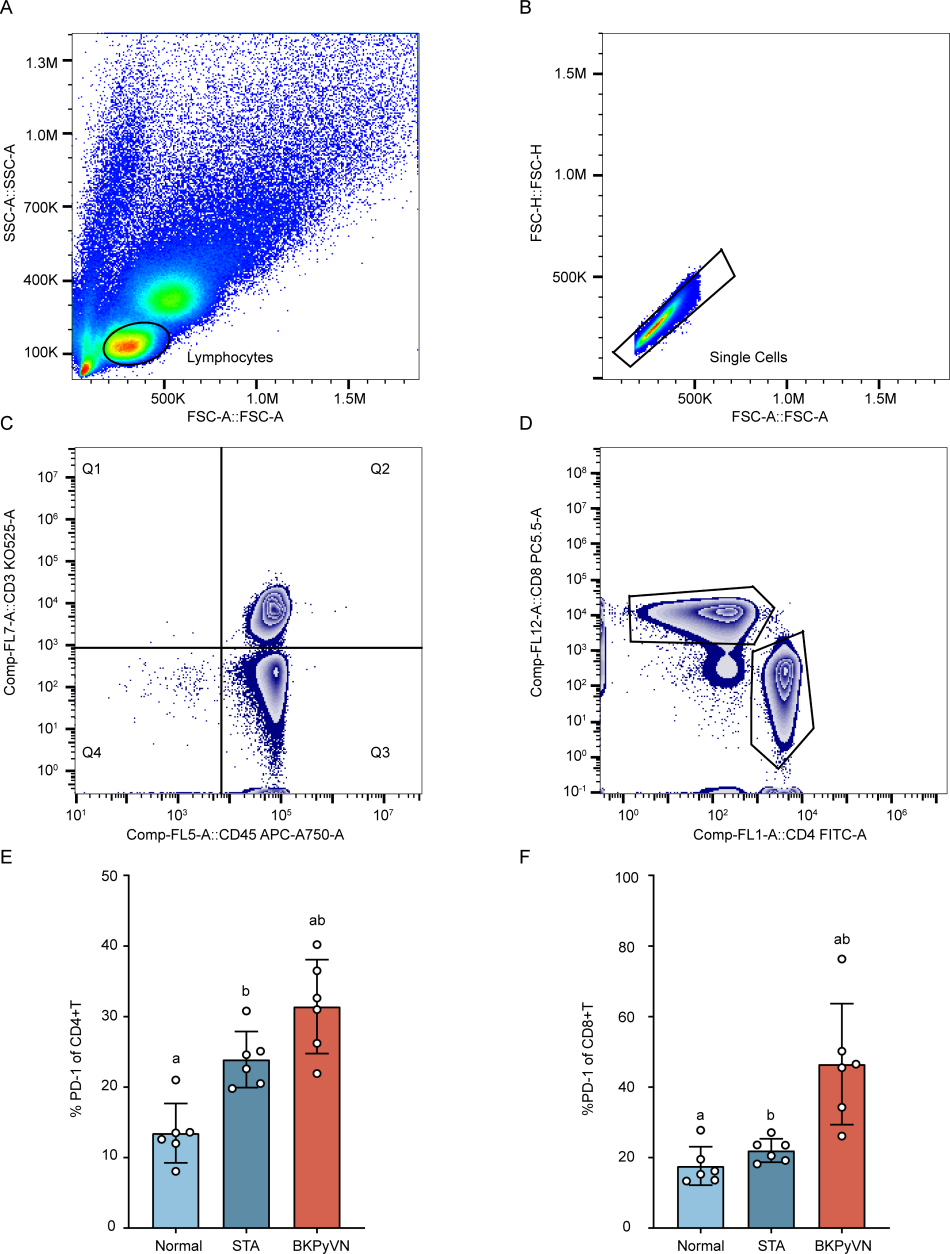
Flow cytometry analysis of T-cell status in BKPyVN samples. (**A–D**) Gating strategy of CD4+ and CD8+ T cells. Percentage of PD-1+ CD4+ T cells (**E**) and PD-1+ CD8+ T cells (**F**) in different groups. Data are presented as the mean ± SD, and significant differences among groups are denoted by different letters (*n* = 6, *P* < 0.05).

We collected 18 blood samples (6 healthy samples, 6 STA samples, and 6 BKPyVN samples) to assess the proportion of exhausted T cells in BKPyVN samples using flow cytometry. The lymphocyte population was initially distinguished using forward corner scatter (FSC-A) and skewed corner scatter (SSC-A) (Fig. 5A). Next, individual cells were defined based on forward scatter area (FSC-A) and height (FSC-H) (Fig. 5B). CD3+ T cells were then circled based on the negative staining control (Fig. 5C), where CD4+ T cells and CD8+ T cells were identified (Fig. 5D). Finally, the proportion of PD-1-positive cells was determined based on the isotype control. The proportion of PD-1-positive cells among CD4+ T cells (Fig. 5E) and CD8+ T cells (Fig. 5F) was significantly higher in the BKPyVN group than in healthy individuals (*P* < 0.05) and the STA group (*P* < 0.05). This finding further demonstrates T-cell exhaustion in the BKPyVN immune microenvironment.

### Regulatory network of cell-cell communication in BKPyVN

**Fig 6:**
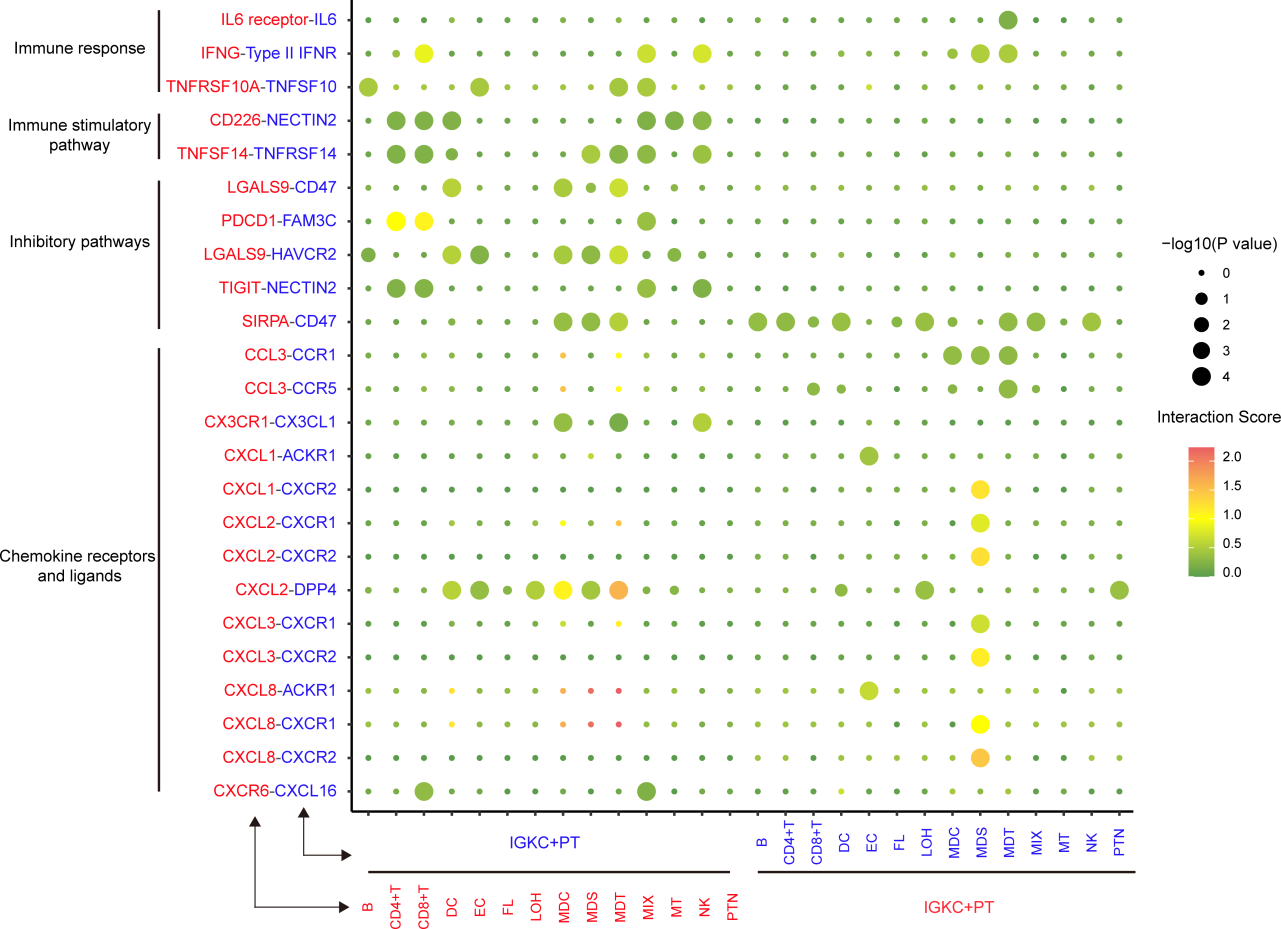
Summary of selected ligand-receptor interactions between IGKC+ PT cells and other subpopulations. Ligands expressed by a given cell population are indicated by a line with a matching color. Interaction scores (ranging from 0.0 to 2.0) are represented by color intensity. *P* values are visualized by dot size.

We used CellPhoneDB (v.2.1.6) to identify interactions between IGKC+ PT cells and other cell subpopulations in BKPyVN samples. To highlight the role of the IGKC+ PT subpopulation, we combined GPX3+ PT and DCXR+ PT into PTN cells. According to CellPhoneDB quantitative analyses (Table S5), PDCD1-FAM3C and TIGIT-NECTIN2 exhibited statistically significant interactions within T|IGKC+ PT cells (*P* < 0.01). The interaction score for PDCD1-FAM3C was 1.016 in the CD4|IGKC+ PT group and 1.121 in the CD8|IGKC+ PT group. The scores for TIGIT-NECTIN2 in the CD4|IGKC+ PT and CD8|IGKC+ PT groups were 0.228 and 0.250, respectively (Fig. 6). These results indicate that the IGKC+ PT subpopulation suppressed the immune response by exhausting CD4+ and CD8+ T cells via PDCD1-FAM3C and TIGIT-NECTIN2 ligand-receptor pairs. To further validate the relationship between IGKC+ PT and T cells, we demonstrated the expression of ligand-receptor pairs associated with T-cell depletion (Fig. S6). Taken together, these findings indicate that the IGKC+ PT subpopulation could induce T-cell exhaustion via PDCD1-FAM3C and TIGIT-NECTIN2.

### Expression profiling and major cell types in BKPyVN urine samples

**Fig 7:**
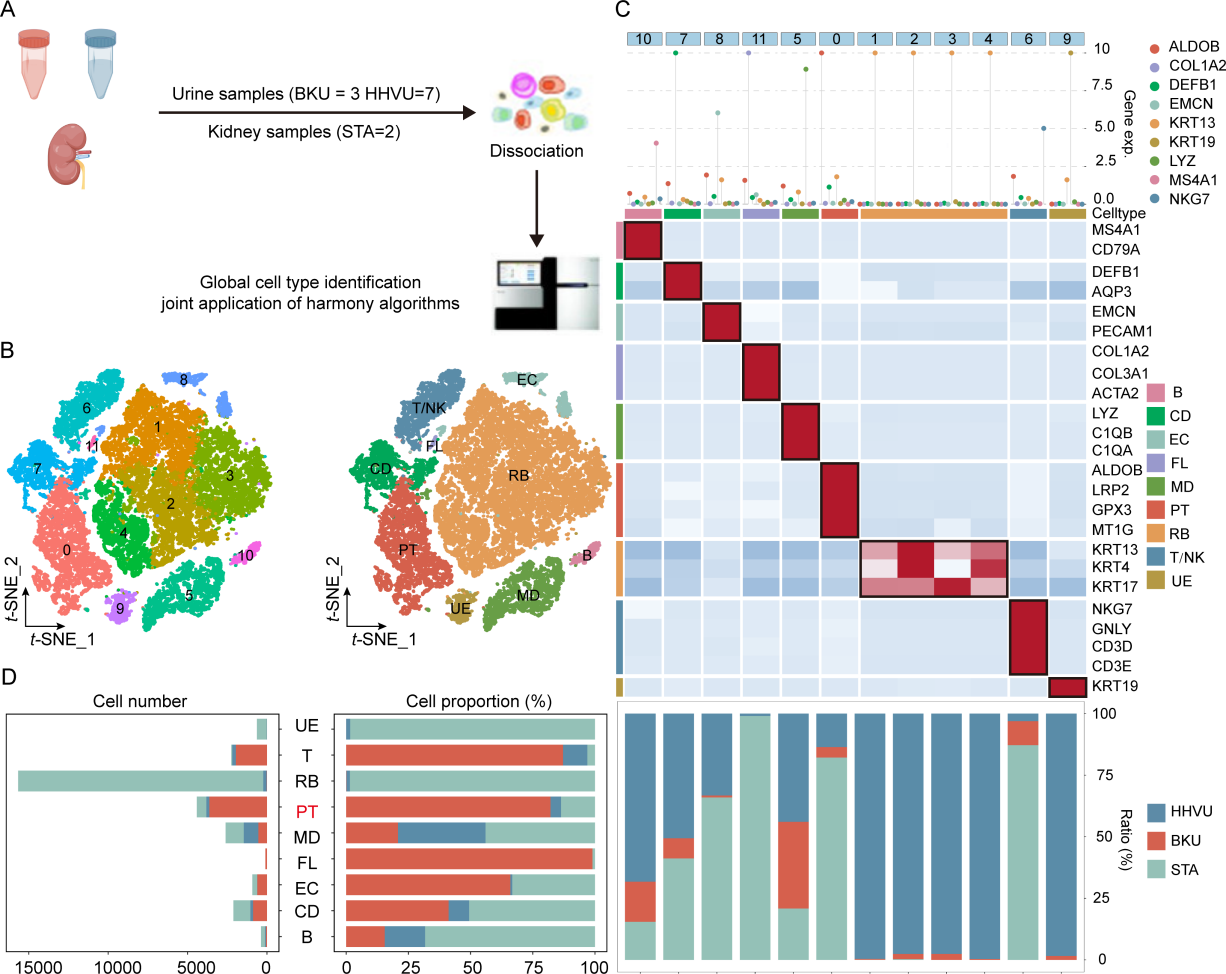
Cell-type identification in BKPyVN urine samples. (**A**) Overview of sample collection and single-cell transcriptomic analysis. (**B**) t-SNE plot displaying 21,845 cells, color-coded by cell clusters or major cell types. (**C**) Three-layered complex heatmap displaying the selected cell marker genes per cluster. (Top) Mean expression of known lineage markers, (middle) group preference of each cluster, and (bottom) relative expression map of known marker genes associated with each cell subpopulation. Mean expression values are centered and scaled from −2 to 2. (**D**) Number and relative proportion of major cell types in different groups.

To confirm the value of the IGKC+ PT subpopulation as a non-invasive diagnostic marker in BKPyVN urine samples, we performed scRNA-seq using urine samples from patients with BKPyVN (BKU, *n* = 3), urine samples from healthy volunteers (HHVU, *n* = 7), and STA kidney samples (*n* = 2) (Fig. 7A). We used the sctransform and harmony integration methods to annotate 11 cell clusters and then identified 9 major cell types based on classical cell-type markers (Fig. S7; Fig. 7B): PT (ALDOB+, LRP2+, GPX3+, and MT1G+), CD (DEFB1+ and AQP3+), FL (CLOL1A2+, COL3A1+, and ACTA2+), EC (EMCN+ and PECAM1+), urothelial cells (KRT19+), bladder epithelial cells (KRT13+, KRT4+, and KRT17+), T/NK (NKG7+, GNLY+, CD3D+, and CD3E+), B (MS4A1 + and CD79A+), and MD (LYZ+, C1QB+, and C1QA+) (Fig. 7C). Notably, the transcriptional signature of PT cells in urine samples (BKU and HHVU) and STA kidney samples was remarkably similar and nearly identical (Fig. 7C, bottom); these PT cells were found in the same cell cluster (Cluster 0), suggesting their presence in urine. A noticeable number of PT cells were present in urine samples from patients with BKPyVN (Fig. 7D). Additionally, we confirmed that the PT cells in urine samples were derived from the kidneys by integrating single-cell transcriptomic data and kidney spatial transcriptomic data using multimodal intersection analysis (Fig. S8; Material S2).

**Fig 8:**
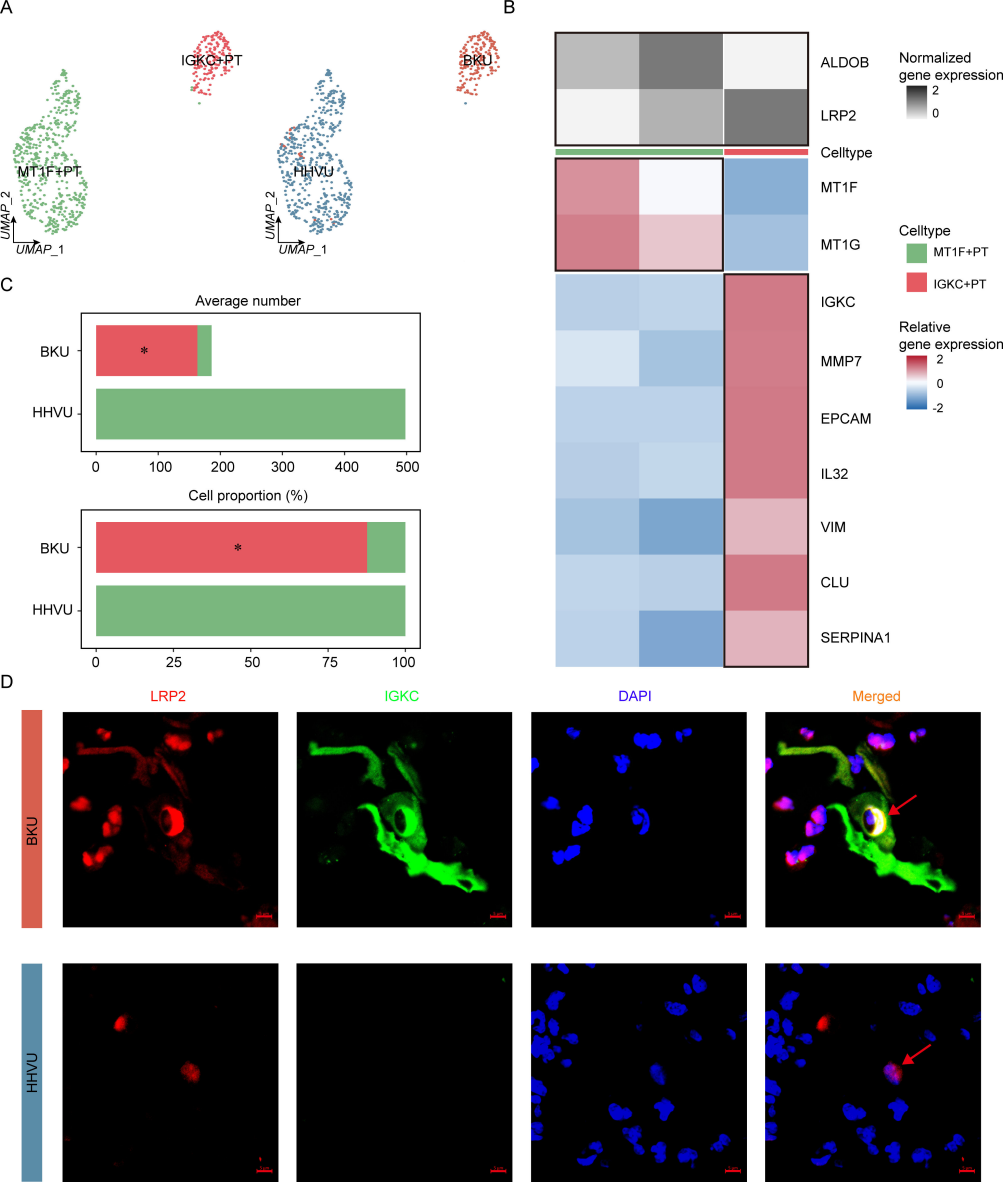
Epithelial-immune dual features of PT cells in BKPyVN urine samples. (**A**) Analysis of cell subpopulations revealed a subpopulation of PT cells with specific dual epithelial-immune characteristics in urine samples of BKPyVN. (**B**) Two-layered complex heatmap indicating the selected canonical markers in each cell subpopulation. (Top) Mean expression of PT canonical markers, (bottom) relative expression map of known marker genes associated with each cell subpopulation. Relative expression values are scaled using mean centering and transformed to a scale from −2 to 2. (**C**) Proportion and average number of cell subpopulations in different groups. (**D**) Immunofluorescence analysis of IGKC+ PT cell subpopulations in urine samples (*n* = 6, scale bar = 5 µm).

Our analysis of 788 urinary PT cells revealed two distinct subpopulations in urine (Fig. 8A). The MT1F+ PT subpopulation was predominantly found in HHVU urine samples, whereas the IGKC+ PT subpopulation was present only in urine samples from patients with BKPyVN (Fig. 8B and C). Immunofluorescence co-staining with antibodies against LRP2 and IGKC revealed double-positive PT cells (IGKC+ PT) in urine samples from patients with BKPyVN but not in HHVU urine samples (Fig. 8D). The proportion of IGKC+ PT cells was significantly associated with the urinary BKPyV viral load (Pearson correlation = 0.99, *P* = 0.008; Table S6). The results indicated that the IGKC+ PT subpopulation present in urine might be useful as a non-invasive diagnostic marker for BKPyVN.

## DISCUSSION

Managing BKPyVN remains a clinical challenge (19, 20). However, understanding its microenvironmental heterogeneity could help identify new therapeutic targets. We demonstrated the presence of epithelial-immune dual features of the IGKC+ PT subpopulation in BKPyVN patients and showed that this subpopulation could contribute to the progression of BKPyVN by exhausting T cells. Our findings suggest that new therapeutic approaches targeting the IGKC+ PT subpopulation could be developed.

Determining the cell-type composition in patients with BKPyVN is crucial for understanding disease progression (21, 22). We observed a significant decrease in the number of PT cells and a significant increase in the number of T cells in the BKPyVN group compared with those in the STA group. This finding aligns with established clinical features of BKPyVN, which include the renal tubular epithelial cell damage and extensive immune cell infiltration (20). We also discovered significant differences in the proportions of PT and T cells between the BKPyVN and STA groups, suggesting that these two cell types play an essential role in BKPyVN.

We identified novel epithelial-immune dual features of the IGKC+ PT subpopulation in the BKPyVN group through PT subpopulation analysis and immunofluorescence co-staining assay. Previous scRNA-seq studies of BKPyV (16, 23) systematically characterized the gene signature of BKPyV-infected PT cells and found that genes in these cells are mainly enriched in cell proliferation and immune signaling pathways, in alignment with our current observations. Specifically, we observed HSP90AA1 as a characteristic gene in the IGKC+ PT subpopulation, and this gene was identified in a previous study as a marker of BKPyV infection in PT cells. The IGKC gene encodes the constant domain of the light chain in antibodies. Initially, this gene was believed to be present only in B cells. However, recent studies have shown that it is also expressed in non-B cells, such as various types of tumor cells (24, 25), central neurons, cells in the placenta, and testis, and mammary epithelial cells during the proleptic phase (26, 27). Previous studies have shown that non-B-cell-expressed IGKC not only functions as a natural antibody (28, 29) but also promotes cell proliferation, cancer development, and metastasis (30), in contrast to B-cell-expressed IGKC. BKPyV replication is known to rely on host cell proliferation (12, 16).

We assessed the association between IGKC+ PT subpopulation and the progression of BKPyVN by analyzing BKPyV reads. However, obtaining renal biopsy specimens that meet the samples requirements of the Smart-seq2 platform is difficult. The combination of BD platform limitations (low sequencing depth) and the small genome size of BKPyV (5.0 kb) results in minimal detection sensitivity. Based on these considerations and an established methodology (31), we assessed viral infection by applying GSVA based on the gene signatures in BKPyV-infected samples reported previously (16). Subsequently, we aimed to clarify the role of IGKC+ PT subpopulation in BKPyVN progression. However, the limited cohort size constrained robust comparisons, while large-scale sequencing was cost prohibitive. As an alternative, we employed a computational deconvolution strategy (21). Given the lack of detailed clinical annotations in published data sets (especially for BKPyVN progression), we could not directly explore the role of IGKC+ PT cells in the progression of BKPyVN. GSVA and GSEA results demonstrated that the IGKC+ PT subpopulation may contribute to BKPyVN progression.

Notably, the gene set in the IGKC+ PT subpopulation that changed significantly with pseudotime was significantly enriched in the immune checkpoint-associated signaling pathway, suggesting that the IGKC+ PT subpopulation, similar to cancer-associated fibroblasts (32, 33), might induce T-cell exhaustion. Subsequently, we demonstrated via T-cell functional analysis and flow cytometry that T cells in the BKPyVN immune microenvironment were in a state of exhaustion. This explains why the large number of infiltrating T cells in the BKPyVN microenvironment does not effectively clear BKPyV (7). Our results suggested that T-cell exhaustion plays a vital role in BKPyVN and indicated that immunotherapeutic approaches targeting T-cell exhaustion could be applied as a BKPyVN treatment strategy. We further demonstrated via cell-cell communication analysis that the IGKC+ PT subpopulation suppressed the immune response by causing CD4+ and CD8+ T-cell exhaustion through the PDCD1-FAM3C and TIGIT-NECTIN2 ligand-receptor pairs. PDCD1-FAM3C and TIGIT-NECTIN2 are previously identified immune checkpoints involved in T-cell depletion (34, 35). Current *in vitro* experiments cannot fully mimic the complex microenvironment of renal cells *in vivo* (36, 37). Moreover, BKPyV is species specific (38), which makes it challenging to validate the detailed molecular mechanisms by which the IGKC+ PT subpopulation regulates T-cell depletion.

To further investigate the potential utility of the IGKC+ PT subpopulation in non-invasive diagnostics, we demonstrated the presence of the IGKC+ PT subpopulation in urine samples from patients with BKPyVN. The high cost of scRNA-seq might limit its clinical applications (39, 40). Developing bioinformatics methods for scRNA-seq (41, 42), such as the deconvolution algorithm used to calculate the proportions of different cell types in urine samples (21, 43, 44), could address this issue. Nevertheless, the deconvolution method requires high-quality single-cell transcriptome data (45). Our study may encourage more researchers to participate in BKPyVN urine single-cell transcriptome research.

In conclusion, we demonstrated that the IGKC+ PT subpopulation promoted BKPyVN progression via T-cell exhaustion. The IGKC+ PT subpopulation might be a promising target for BKPyVN treatment and a novel non-invasive diagnostic marker.

## MATERIALS AND METHODS

### Tissue and urine sample collection

We performed scRNA-seq on three BKPyVN biopsy tissues and two STA samples. Additionally, we profiled the single-cell transcriptome in three urine samples from patients with BKPyVN and seven urine samples from healthy volunteers to identify potential non-invasive biomarkers. The detailed clinical characteristics are summarized in Table S7.

### scRNA-seq and data processing

The kidney biopsy tissues were processed as described in prior studies (46, 47), and established protocols were used to analyze the urine samples (48, 49). Briefly, kidney samples were minced and then digested with an enzyme to obtain single-cell suspensions (Supplemental Material S3). We used the BD Rhapsody Whole Transcriptome assay analysis pipeline to acquire raw gene expression matrices. Subsequent analyses were performed using Seurat (v.4.0.2). The SCT method (v.0.3.5) was used for normalization. The R package “Harmony” (v.0.1.1) was used for batching correction. The following criteria were applied: cells with >500 genes, <4,000 genes, and <30% mitochondrial gene expression in unique molecular identifier (UMI) counts (for urine samples, <60% mitochondrial gene expression in UMI counts) (33). We employed a graph-based clustering approach with a 0.2 resolution to detect cell clusters and subsequently identified major cell types using canonical marker genes from prior studies (46, 47, 50, 51).

### Correlation with public data sets

The microarray cohort contained gene expression profiles associated with BKPyVN (*n* = 28) and control groups (*n* = 109) that were downloaded from the GEO database (GSE75693, GSE47199, and GSE72925; accessed on 6 June 2020) (52–54). The ComBat algorithm (55) was used to eliminate batch effects. To evaluate the relative abundance of each cell type identified in the present study, MuSiC (v.0.1.1) (42) was used, along with the method of pre-grouping cell types. scRNA-seq of kidney tissue samples served as a reference for estimating cell-type proportions in bulk data. Subsequently, we summarized the set of genes related to the progression of BKPyVN based on the Molecular Signatures Database (56) and literature reports (16). Then, the BKPyVN data cohort was divided into high (50%) and low (50%) groups according to the relative cell abundance of the IGKC+ PT subpopulation. Finally, we employed GSEA to explore the relationship between the abundance of IGKC+ PT cell clusters and the progression of BKPyVN. According to the 2019 guidelines of the American Society of Transplantation (1), the progression of BKPyVN primarily relies on an evaluation of inflammation, fibrosis, and BKPyV infection. Thus, we obtained gene sets related to inflammation, fibrosis, and BKPyV infection from hallmark gene sets and previous studies. The inflammation included INFLAMMATORY RESPONSE, INTERFERON ALPHA RESPONSE, and INTERFERON GAMMA RESPONSE. The fibrosis contained EPITHELIAL MESENCHYMAL TRANSITION. The BKPyV infection comprised E2F TARGETS and BKPyV REPLICATION. Previous studies have shown that BKPyV interacts with the E2F TARGETS signaling pathway to hijack the host cell cycle and promote viral replication (57–59). The BKPyV REPLICATION is a gene signature of BKPyV-infected PT cells, as identified in a previous study (16).

### Pseudotime trajectory analysis

We used Monocle2 (v.2.18.0) (60) to investigate the mechanisms by which PT cells promoted disease progression. Briefly, we reconstructed the cellular differentiation trajectory of PT cells and identified the genes involved in the transition from a normal state to a disease state. We employed the “differentGeneTest” function to select signature genes. Then, we reduced the dimensionality of the data using Discriminative Dimensionality Reduction via Learning a Tree and ordered the cells in pseudotime. Finally, we performed KEGG pathway enrichment analysis using KOBAS (v.3.0) (61).

### T-cell functional analysis

To evaluate the functional status of CD8 +and CD4+T cells, a set of genes associated with cytotoxicity (NKG7, PRF1, GZMA, GZMB, GZMK, IFNG, CCL4, and CST7) and exhaustion (LAG3, TIGIT, PDCD1 [also known as PD1], HAVCR2, and CTLA4) were used to calculate cytotoxicity and exhaustion scores, based on previous studies (6, 62).

### Cell-cell interaction analysis

We used CellPhoneDB (v.2.1.6) (63) to establish a cell-cell communication network within 15 identified cell types, using known ligand-receptor pairs. Only receptors and ligands expressed in more than 10% of the cells were included.

### Immunofluorescence analysis

Multiplex staining was performed using the PANO 7-plex IHC kit (Panovue, Beijing, China) on 12 samples (6 BKPyVN and 6 STA) according to the manufacturer’s instructions. The following primary antibodies were used: anti-LRP2 (1:200; Abcam, A5228) and anti-IGKC (1:4,000; Abcam, ZM-0044). To obtain multispectral images, five randomly selected fields on each stained slide were scanned using the Mantra System at ×200 magnification.

### Flow cytometry

We obtained 18 samples (6 BKPyVN, 6 STA, and 6 healthy samples) to determine the state of T cells in patients with BKPyVN. The following antibodies were used: anti- CD45 (1:300; BD, 566563), anti- CD3 (1:100; BD, 555482), and anti- CD4 (1:500; BD, 562799). Anti-CD8 (1:100; BD, 555482) and anti-CD279 (PD-1) (1:200; BD, 555335) were used to confirm the subpopulations of T-cell lineage. Data analysis was performed in FlowJo (v.10).

## Data Availability

The scRNA-seq data in the study have been uploaded to the National Genomics Data Center (accession number: HRA002472, https://ngdc.cncb.ac.cn/gsa-human/s/1I8ts729). Spatial transcriptome data were obtained from the GEO data set (GSM5224978).

## SUPPLEMENTAL MATERIAL

Supplemental material jvi.01394-25-s0001.docx Material S1 to S3; Fig. S1 to S8.

Supplemental tables jvi.01394-25-s0002.xlsx Tables S1 to S7.

ASM does not own the copyrights to Supplemental Material that may be linked to, or accessed through, an article. The authors have granted ASM a non-exclusive, world-wide license to publish the Supplemental Material files. Please contact the corresponding author directly for reuse.

## References

[1] Hirsch HH, Randhawa PS, ASTIDCo P. 2019. BK polyomavirus in solid organ transplantation-Guidelines from the American society of transplantation infectious diseases community of practice. Clin Transplant 33:e13528. doi:10.1111/ctr.13528 30859620

[2] Furmaga J, Kowalczyk M, Zapolski T, Furmaga O, Krakowski L, Rudzki G, Jaroszyński A, Jakubczak A. 2021. BK polyomavirus-biology, genomic variation and diagnosis. Viruses 13:1502. doi:10.3390/v13081502 34452367 PMC8402805

[3] Mayr M, Nickeleit V, Hirsch HH, Dickenmann M, Mihatsch MJ, Steiger J. 2001. Polyomavirus BK nephropathy in a kidney transplant recipient: critical issues of diagnosis and management. Am J Kidney Dis 38:E13. doi:10.1053/ajkd.2001.26917 11532715

[4] Hirsch HH, Babel N, Comoli P, Friman V, Ginevri F, Jardine A, Lautenschlager I, Legendre C, Midtvedt K, Muñoz P, Randhawa P, Rinaldo CH, Wieszek A, ESCMID Study Group of Infection in Compromised Hosts. 2014. European perspective on human polyomavirus infection, replication and disease in solid organ transplantation. Clin Microbiol Infect 20 Suppl 7:74–88. doi:10.1111/1469-0691.12538 24476010

[5] Fu J, Akat KM, Sun Z, Zhang W, Schlondorff D, Liu Z, Tuschl T, Lee K, He JC. 2019. Single-cell RNA profiling of glomerular cells shows dynamic changes in experimental diabetic kidney disease. J Am Soc Nephrol 30:533–545. doi:10.1681/ASN.2018090896 30846559 PMC6442341

[6] Jin S, Li R, Chen M-Y, Yu C, Tang L-Q, Liu Y-M, Li J-P, Liu Y-N, Luo Y-L, Zhao Y, et al.. 2020. Single-cell transcriptomic analysis defines the interplay between tumor cells, viral infection, and the microenvironment in nasopharyngeal carcinoma. Cell Res 30:950–965. doi:10.1038/s41422-020-00402-8 32901110 PMC7784966

[7] Kotla SK, Kadambi PV, Hendricks AR, Rojas R. 2021. BK polyomavirus-pathogen, paradigm and puzzle. Nephrol Dial Transplant 36:587–593. doi:10.1093/ndt/gfz273 31891401

[8] Stervbo U, Nienen M, Weist BJD, Kuchenbecker L, Hecht J, Wehler P, Westhoff TH, Reinke P, Babel N. 2019. BKV clearance time correlates with exhaustion state and T-cell receptor repertoire shape of BKV-specific T-cells in renal transplant patients. Front Immunol 10:767. doi:10.3389/fimmu.2019.00767 31024575 PMC6468491

[9] Wilhelm M, Kaur A, Geng A, Wernli M, Hirsch HH. 2025. Donor variability and PD-1 expression limit BK polyomavirus-specific T-cell function and therapy. Transplantation 109:1526–1539. doi:10.1097/TP.0000000000005399 40200394 PMC12366737

[10] Moriyama T, Marquez JP, Wakatsuki T, Sorokin A. 2007. Caveolar endocytosis is critical for BK virus infection of human renal proximal tubular epithelial cells. J Virol 81:8552–8562. doi:10.1128/JVI.00924-07 17553887 PMC1951339

[11] Low J, Humes HD, Szczypka M, Imperiale M. 2004. BKV and SV40 infection of human kidney tubular epithelial cells in vitro. Virology (Auckl) 323:182–188. doi:10.1016/j.virol.2004.03.027 15193914

[12] Yang F, Chen X, Zhang H, Zhao G-D, Yang H, Qiu J, Meng S, Wu P, Tao L, Wang Q, Huang G. 2023. Single-cell transcriptome identifies the renal cell type tropism of human BK polyomavirus. Int J Mol Sci 24:1330. doi:10.3390/ijms24021330 36674845 PMC9861348

[13] Starke A, Lindenmeyer MT, Segerer S, Neusser MA, Rüsi B, Schmid DM, Cohen CD, Wüthrich RP, Fehr T, Waeckerle-Men Y. 2010. Renal tubular PD-L1 (CD274) suppresses alloreactive human T-cell responses. Kidney Int 78:38–47. doi:10.1038/ki.2010.97 20393451

[14] Li H, Dixon EE, Wu H, Humphreys BD. 2022. Comprehensive single-cell transcriptional profiling defines shared and unique epithelial injury responses during kidney fibrosis. Cell Metab 34:1977–1998. doi:10.1016/j.cmet.2022.09.026 36265491 PMC9742301

[15] Li R, Ferdinand JR, Loudon KW, Bowyer GS, Laidlaw S, Muyas F, Mamanova L, Neves JB, Bolt L, Fasouli ES, et al.. 2022. Mapping single-cell transcriptomes in the intra-tumoral and associated territories of kidney cancer. Cancer Cell 40:1583–1599. doi:10.1016/j.ccell.2022.11.001 36423636 PMC9767677

[16] An P, Cantalupo PG, Zheng W, Sáenz-Robles MT, Duray AM, Weitz D, Pipas JM. 2021. Single-cell transcriptomics reveals a heterogeneous cellular response to BK virus infection. J Virol 95:e02237-20. doi:10.1128/JVI.02237-20 33361432 PMC8094954

[17] Liang B, Tikhanovich I, Nasheuer HP, Folk WR. 2012. Stimulation of BK virus DNA replication by NFI family transcription factors. J Virol 86:3264–3275. doi:10.1128/JVI.06369-11 22205750 PMC3302295

[18] Yang JF, You J. 2020. Regulation of polyomavirus transcription by viral and cellular factors. Viruses 12:10. doi:10.3390/v12101072 PMC7601649 32987952

[19] Kant S, Dasgupta A, Bagnasco S, Brennan DC. 2022. BK virus nephropathy in kidney transplantation: a state-of-the-art review. Viruses 14:1616. doi:10.3390/v14081616 35893681 PMC9330039

[20] Imlay H, Baum P, Brennan DC, Hanson KE, Hodges MR, Hodowanec AC, Komatsu TE, Ljungman P, Miller V, Natori Y, Nickeleit V, O’Rear J, Pikis A, Randhawa PS, Sawinski D, Singh HK, Westman G, Limaye AP. 2022. Consensus definitions of BK polyomavirus nephropathy in renal transplant recipients for clinical trials. Clin Infect Dis 75:1210–1216. doi:10.1093/cid/ciac071 35100619 PMC9525067

[21] Wang X, Park J, Susztak K, Zhang NR, Li M. 2019. Bulk tissue cell type deconvolution with multi-subject single-cell expression reference. Nat Commun 10:380. doi:10.1038/s41467-018-08023-x 30670690 PMC6342984

[22] Chung J-J, Goldstein L, Chen Y-JJ, Lee J, Webster JD, Roose-Girma M, Paudyal SC, Modrusan Z, Dey A, Shaw AS. 2020. Single-cell transcriptome profiling of the kidney glomerulus identifies key cell types and reactions to injury. J Am Soc Nephrol 31:2341–2354. doi:10.1681/ASN.2020020220 32651223 PMC7609001

[23] Weissbach FH, Follonier OM, Schmid S, Leuzinger K, Schmid M, Hirsch HH. 2024. Single-cell RNA-sequencing of BK polyomavirus replication in primary human renal proximal tubular epithelial cells identifies specific transcriptome signatures and a novel mitochondrial stress pattern. J Virol 98:e0138224. doi:10.1128/jvi.01382-24 39513696 PMC11657676

[24] Sheng Z, Liu Y, Qin C, Liu Z, Yuan Y, Hu F, Du Y, Yin H, Qiu X, Xu T. 2016. IgG is involved in the migration and invasion of clear cell renal cell carcinoma. J Clin Pathol 69:497–504. doi:10.1136/jclinpath-2015-202881 26519488 PMC4893138

[25] Qiu X, Zhu X, Zhang L, Mao Y, Zhang J, Hao P, Li G, Lv P, Li Z, Sun X, Wu L, Zheng J, Deng Y, Hou C, Tang P, Zhang S, Zhang Y. 2003. Human epithelial cancers secrete immunoglobulin G with unidentified specificity to promote growth and survival of tumor cells. Cancer Res 63:6488–6495. 14559841

[26] Huang J, Sun X, Mao Y, Zhu X, Zhang P, Zhang L, Du J, Qiu X. 2008. Expression of immunoglobulin gene with classical V-(D)-J rearrangement in mouse brain neurons. Int J Biochem Cell Biol 40:1604–1615. doi:10.1016/j.biocel.2007.12.004 18243769

[27] Li J, Korteweg C, Qiu Y, Luo J, Chen Z, Huang G, Li W, Gu J. 2014. Two ultrastructural distribution patterns of immunoglobulin G in human placenta and functional implications. Biol Reprod 91:128. doi:10.1095/biolreprod.114.122614 25273527

[28] Jiang D, Ge J, Liao Q, Ma J, Liu Y, Huang J, Wang C, Xu W, Zheng J, Shao W, Lee G, Qiu X. 2015. IgG and IgA with potential microbial-binding activity are expressed by normal human skin epidermal cells. Int J Mol Sci 16:2574–2590. doi:10.3390/ijms16022574 25625513 PMC4346852

[29] Shao W, Hu F, Ma J, Zhang C, Liao Q, Zhu Z, Liu E, Qiu X. 2016. Epithelial cells are a source of natural IgM that contribute to innate immune responses. Int J Biochem Cell Biol 73:19–29. doi:10.1016/j.biocel.2016.01.017 26820901

[30] Tang J, Zhang J, Liu Y, Liao Q, Huang J, Geng Z, Xu W, Sheng Z, Lee G, Zhang Y, Chen J, Zhang L, Qiu X. 2018. Lung squamous cell carcinoma cells express non-canonically glycosylated IgG that activates integrin-FAK signaling. Cancer Lett 430:148–159. doi:10.1016/j.canlet.2018.05.024 29778566

[31] Marjanovic ND, Hofree M, Chan JE, Canner D, Wu K, Trakala M, Hartmann GG, Smith OC, Kim JY, Evans KV, et al.. 2020. Emergence of a high-plasticity cell state during lung cancer evolution. Cancer Cell 38:229–246. doi:10.1016/j.ccell.2020.06.012 32707077 PMC7745838

[32] Li X, Sun Z, Peng G, Xiao Y, Guo J, Wu B, Li X, Zhou W, Li J, Li Z, Bai C, Zhao L, Han Q, Zhao RC, Wang X. 2022. Single-cell RNA sequencing reveals a pro-invasive cancer-associated fibroblast subgroup associated with poor clinical outcomes in patients with gastric cancer. Theranostics 12:620–638. doi:10.7150/thno.60540 34976204 PMC8692898

[33] Chen Z, Zhou L, Liu L, Hou Y, Xiong M, Yang Y, Hu J, Chen K. 2020. Single-cell RNA sequencing highlights the role of inflammatory cancer-associated fibroblasts in bladder urothelial carcinoma. Nat Commun 11:5077. doi:10.1038/s41467-020-18916-5 33033240 PMC7545162

[34] Breuer K, Foroushani AK, Laird MR, Chen C, Sribnaia A, Lo R, Winsor GL, Hancock REW, Brinkman FSL, Lynn DJ. 2013. InnateDB: systems biology of innate immunity and beyond--recent updates and continuing curation. Nucleic Acids Res 41:D1228–D1233. doi:10.1093/nar/gks1147 23180781 PMC3531080

[35] Garcia-Alonso L, Lorenzi V, Mazzeo CI, Alves-Lopes JP, Roberts K, Sancho-Serra C, Engelbert J, Marečková M, Gruhn WH, Botting RA, Li T, Crespo B, van Dongen S, Kiselev VY, Prigmore E, Herbert M, Moffett A, Chédotal A, Bayraktar OA, Surani A, Haniffa M, Vento-Tormo R. 2022. Single-cell roadmap of human gonadal development. Nature 607:540–547. doi:10.1038/s41586-022-04918-4 35794482 PMC9300467

[36] An P, Sáenz Robles MT, Duray AM, Cantalupo PG, Pipas JM. 2019. Human polyomavirus BKV infection of endothelial cells results in interferon pathway induction and persistence. PLoS Pathog 15:e1007505. doi:10.1371/journal.ppat.1007505 30620752 PMC6338385

[37] Popik W, Khatua AK, Fabre NF, Hildreth JEK, Alcendor DJ. 2019. BK virus replication in the glomerular vascular unit: implications for BK virus associated nephropathy. Viruses 11:583. doi:10.3390/v11070583 31252545 PMC6669441

[38] Barth H, Solis M, Kack-Kack W, Soulier E, Velay A, Fafi-Kremer S. 2016. In vitro and in vivo models for the study of human polyomavirus infection. Viruses 8:292. doi:10.3390/v8100292 27782080 PMC5086624

[39] Suvà ML, Tirosh I. 2019. Single-cell RNA sequencing in cancer: lessons learned and emerging challenges. Mol Cell 75:7–12. doi:10.1016/j.molcel.2019.05.003 31299208

[40] Balzer MS, Rohacs T, Susztak K. 2022. How many cell types are in the kidney and what do they do? Annu Rev Physiol 84:507–531. doi:10.1146/annurev-physiol-052521-121841 34843404 PMC9233501

[41] Charytonowicz D, Brody R, Sebra R. 2023. Interpretable and context-free deconvolution of multi-scale whole transcriptomic data with UniCell deconvolve. Nat Commun 14:1350. doi:10.1038/s41467-023-36961-8 36906603 PMC10008582

[42] Fan J, Lyu Y, Zhang Q, Wang X, Li M, Xiao R. 2022. MuSiC2: cell-type deconvolution for multi-condition bulk RNA-seq data. Brief Bioinform 23:bbac430. doi:10.1093/bib/bbac430 36208175 PMC9677503

[43] Dong M, Thennavan A, Urrutia E, Li Y, Perou CM, Zou F, Jiang Y. 2021. SCDC: bulk gene expression deconvolution by multiple single-cell RNA sequencing references. Brief Bioinform 22:416–427. doi:10.1093/bib/bbz166 31925417 PMC7820884

[44] Baron M, Veres A, Wolock SL, Faust AL, Gaujoux R, Vetere A, Ryu JH, Wagner BK, Shen-Orr SS, Klein AM, Melton DA, Yanai I. 2016. A single-cell transcriptomic map of the human and mouse pancreas reveals inter- and intra-cell population structure. Cell Syst 3:346–360. doi:10.1016/j.cels.2016.08.011 27667365 PMC5228327

[45] Im Y, Kim Y. 2023. A comprehensive overview of RNA deconvolution methods and their application. Mol Cells 46:99–105. doi:10.14348/molcells.2023.2178 36859474 PMC9982058

[46] Liu Y, Hu J, Liu D, Zhou S, Liao J, Liao G, Yang S, Guo Z, Li Y, Li S, Chen H, Guo Y, Li M, Fan L, Li L, Lin A, Zhao M. 2020. Single-cell analysis reveals immune landscape in kidneys of patients with chronic transplant rejection. Theranostics 10:8851–8862. doi:10.7150/thno.48201 32754283 PMC7392010

[47] Wu H, Malone AF, Donnelly EL, Kirita Y, Uchimura K, Ramakrishnan SM, Gaut JP, Humphreys BD. 2018. Single-cell transcriptomics of a human kidney allograft biopsy specimen defines a diverse inflammatory response. J Am Soc Nephrol 29:2069–2080. doi:10.1681/ASN.2018020125 29980650 PMC6065085

[48] Abedini A, Zhu YO, Chatterjee S, Halasz G, Devalaraja-Narashimha K, Shrestha R, S Balzer M, Park J, Zhou T, Ma Z, Sullivan KM, Hu H, Sheng X, Liu H, Wei Y, Boustany-Kari CM, Patel U, Almaani S, Palmer M, Townsend R, Blady S, Hogan J, Morton L, Susztak K, TRIDENT Study Investigators. 2021. Urinary single-cell profiling captures the cellular diversity of the kidney. J Am Soc Nephrol 32:614–627. doi:10.1681/ASN.2020050757 33531352 PMC7920183

[49] Wang Y, Zhao Y, Zhao Z, Li D, Nie H, Sun Y, Feng X, Zhang T, Ma Y, Nie J, Cai G, Chen X, Zuo W. 2021. Single-cell RNA-seq analysis identified kidney progenitor cells from human urine. Protein Cell 12:305–312. doi:10.1007/s13238-020-00816-5 33420958 PMC8018990

[50] Wilson PC, Wu H, Kirita Y, Uchimura K, Ledru N, Rennke HG, Welling PA, Waikar SS, Humphreys BD. 2019. The single-cell transcriptomic landscape of early human diabetic nephropathy. Proc Natl Acad Sci USA 116:19619–19625. doi:10.1073/pnas.1908706116 31506348 PMC6765272

[51] Liao J, Yu Z, Chen Y, Bao M, Zou C, Zhang H, Liu D, Li T, Zhang Q, Li J, Cheng J, Mo Z. 2020. Single-cell RNA sequencing of human kidney. Sci Data 7:4. doi:10.1038/s41597-019-0351-8 31896769 PMC6940381

[52] Sigdel TK, Gao Y, He J, Wang A, Nicora CD, Fillmore TL, Shi T, Webb-Robertson B-J, Smith RD, Qian W-J, Salvatierra O, Camp DG 2nd, Sarwal MM. 2016. Mining the human urine proteome for monitoring renal transplant injury. Kidney Int 89:1244–1252. doi:10.1016/j.kint.2015.12.049 27165815 PMC5221536

[53] Lubetzky M, Bao Y, Ó Broin P, Marfo K, Ajaimy M, Aljanabi A, de Boccardo G, Golden A, Akalin E. 2014. Genomics of BK viremia in kidney transplant recipients. Transplantation 97:451–456. doi:10.1097/01.TP.0000437432.35227.3e 24310299

[54] Sigdel T, Nguyen M, Liberto J, Dobi D, Junger H, Vincenti F, Laszik Z, Sarwal MM. 2019. Assessment of 19 genes and validation of CRM gene panel for quantitative transcriptional analysis of molecular rejection and inflammation in archival kidney transplant biopsies. Front Med (Lausanne) 6:213. doi:10.3389/fmed.2019.00213 31632976 PMC6781675

[55] Zhang Y, Parmigiani G, Johnson WE. 2020. ComBat-seq: batch effect adjustment for RNA-seq count data. NAR Genom Bioinform 2:lqaa078. doi:10.1093/nargab/lqaa078 33015620 PMC7518324

[56] Liberzon A, Birger C, Thorvaldsdóttir H, Ghandi M, Mesirov JP, Tamayo P. 2015. The Molecular Signatures Database (MSigDB) hallmark gene set collection. Cell Syst 1:417–425. doi:10.1016/j.cels.2015.12.004 26771021 PMC4707969

[57] Emanuele MJ, Enrico TP, Mouery RD, Wasserman D, Nachum S, Tzur A. 2020. Complex cartography: regulation of E2F transcription factors by cyclin F and ubiquitin. Trends Cell Biol 30:640–652. doi:10.1016/j.tcb.2020.05.002 32513610 PMC7859860

[58] Needham JM, Perritt SE, Thompson SR. 2024. Single-cell analysis reveals host S phase drives large T antigen expression during BK polyomavirus infection. PLoS Pathog 20:e1012663. doi:10.1371/journal.ppat.1012663 39636788 PMC11620372

[59] Caller LG, Davies CTR, Antrobus R, Lehner PJ, Weekes MP, Crump CM. 2019. Temporal proteomic analysis of BK polyomavirus infection reveals virus-induced G_2_ arrest and highly effective evasion of innate immune sensing. J Virol 93:e00595-19. doi:10.1128/JVI.00595-19 31142673 PMC6675895

[60] Qiu X, Hill A, Packer J, Lin D, Ma YA, Trapnell C. 2017. Single-cell mRNA quantification and differential analysis with Census. Nat Methods 14:309–315. doi:10.1038/nmeth.4150 28114287 PMC5330805

[61] Bu D, Luo H, Huo P, Wang Z, Zhang S, He Z, Wu Y, Zhao L, Liu J, Guo J, Fang S, Cao W, Yi L, Zhao Y, Kong L. 2021. KOBAS-i: intelligent prioritization and exploratory visualization of biological functions for gene enrichment analysis. Nucleic Acids Res 49:W317–W325. doi:10.1093/nar/gkab447 34086934 PMC8265193

[62] Lee H-O, Hong Y, Etlioglu HE, Cho YB, Pomella V, Van den Bosch B, Vanhecke J, Verbandt S, Hong H, Min J-W, et al.. 2020. Lineage-dependent gene expression programs influence the immune landscape of colorectal cancer. Nat Genet 52:594–603. doi:10.1038/s41588-020-0636-z 32451460

[63] Garcia-Alonso L, Handfield L-F, Roberts K, Nikolakopoulou K, Fernando RC, Gardner L, Woodhams B, Arutyunyan A, Polanski K, Hoo R, et al.. 2021. Mapping the temporal and spatial dynamics of the human endometrium in vivo and in vitro. Nat Genet 53:1698–1711. doi:10.1038/s41588-021-00972-2 34857954 PMC8648563

