# Supplementary material for: Single-cell RNA sequencing highlights the role of epithelial-immune dual features of proximal tubule cells in BK polyomavirus nephropathy
Source: J Virol. 2025 Oct 7;99(11):e01394-25. doi: 10.1128/jvi.01394-25 (PMC12645914; doi:10.1128/jvi.01394-25)
Supplement: Supplemental material — Material S1 to S3; Fig. S1 to S8. [file jvi.01394-25-s0001.docx]

**Material S1. Cell type specific regulon activity analysis**

Transcription factor regulatory networks at single-cell resolution were constructed using pySCENIC (v0.11.2), a Python implementation of the SCENIC pipeline^1^. Briefly, regulons were first inferred based on gene expression profile correlations across cells using the GRNBoost2 algorithm. Second, transcription factor motif analysis was performed using the cisTARGET databases (https://resources.aertslab.org/cistarget/) to identify candidate direct-binding targets. Third, regulon activity was quantified using AUCell to compute enrichment scores for target gene sets within each cell. Finally, cell-type-specific regulons were identified according to the regulon specificity score (RSS), calculated using the Jensen-Shannon divergence (JSD) algorithm. SEEK analysis was done to select datasets in which the transcription factor and its target genes within a regulon are co-expressed. We queried the titles for public human datasets for enrichment of cell-type-specific terms, with the assumption that functionally related genes tend to be co-expressed in the corresponding cell types.

**Material S2 Multimodal intersection analysis of stRNA-seq**

Firstly, we obtained kidney tissue spatial transcriptomic data from the public Gene Expression Omnibus (GEO) database (GSM5224978)^2^. Upon importing the data, normalization and standardization were performed using SCTransform (v 0.3.5). Clustering analysis was conducted using 10 principal components with a resolution threshold of 0.5. Subsequently, the RunUMAP function was used for nonlinear dimensionality reduction, projecting the data into a low-dimensional space. Finally, cell type annotation was performed by integrating marker genes and tissue characteristics reported in the previous study^2^.

Subsequently, we identified marker genes for distinct cell types in both stRNA-seq of kidney tissue and scRNA-seq of urine using the FindAllMarkers function, with thresholds set to min.pct = 0.3 and logfc.threshold = 0.25.

Finally, we employed multimodal intersection analysis (MIA) to assess the localization potential of proximal tubule epithelial cells derived from urine single-cell transcriptomes within the renal proximal tubule. Based on previous study^3^, we evaluated the significance of the overlap between STRNS-seq genes and cell type marker genes using the hypergeometric cumulative distribution. All genes served as the background for P-value computation. For enrichment, it is determined using -log₁₀(P), whereas depletion is calculated based on -log₁₀(1-P).

**Material S3. Single-cell dissociation**

Single-cell RNA-seq experiment was performed by experimental personnel in the laboratory of NovelBio Bio-Pharm Technology Co.,Ltd. The tissues of renal biopsy were surgically removed and kept in MACS Tissue Storage Solution (NO: 130-100-008, Miltenyi Biotec) until processing. The tissue samples were processed as described below. Briefly, samples were first washed with phosphate-buffered saline (PBS), minced into small pieces (approximately 1mm^3^) on ice and enzymatically digested with 200 U/mL collagenase I (Worthington),50 U/mL collagenase IV (Worthington)and 30 U/mL DNase I (Worthington) for 25 min at 37°C, with agitation. After digestion, samples were sieved through a 40µm cell strainer, and centrifuged at 500g for 8 min. After the supernatant was removed, the pelleted cells were suspended in red blood cell lysis buffer (NO: 130-094-183, Miltenyi Biotec) to lyse red blood cells. After washing with RPMI-1640(Gibco), the cell pellets were re-suspended in sample buffer (BD Biosciences). Dissociated single cells were then stained for viability assessment using Calcein-AM (NO: C1430, Thermo Fisher Scientific) and Draq7 (NO: 564904, BD Biosciences).


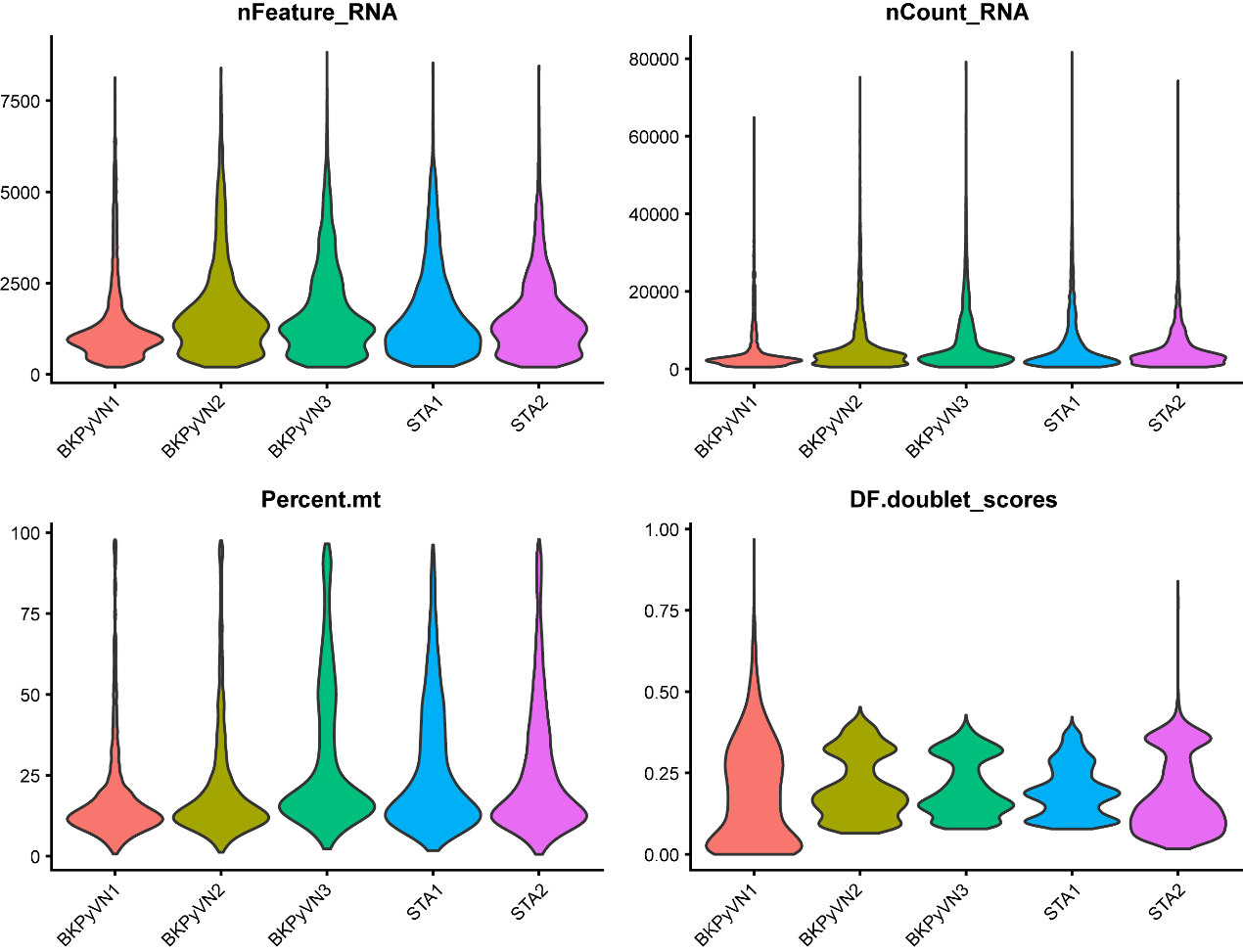


**Fig. S1 Before quality control, the number of genes, UMIs, percentage of mitochondrial genes, and doublet scores in each sample.**


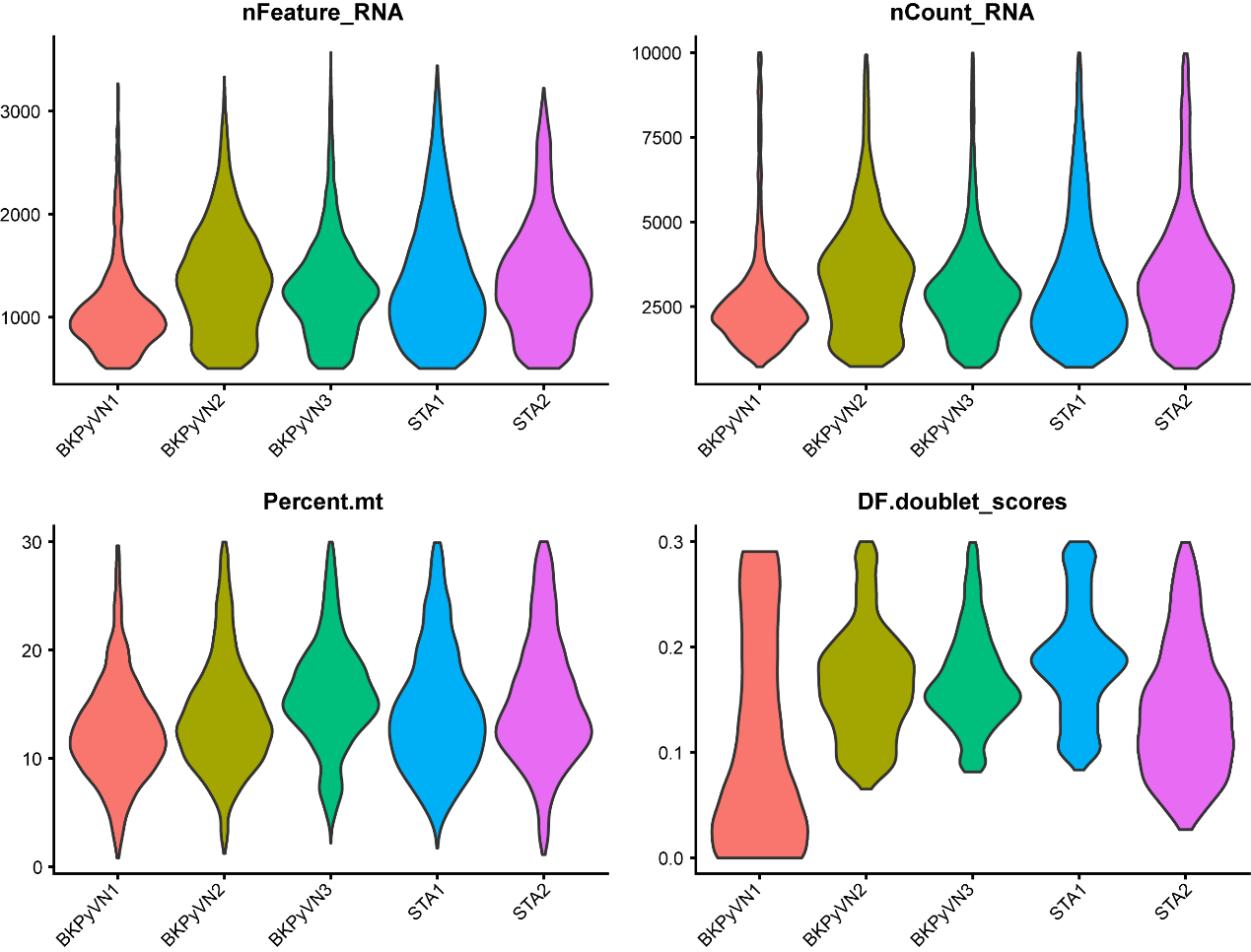


**Fig. S2 After quality control, the number of genes, UMIs, percentage of mitochondrial genes, and doublet scores in each sample.**


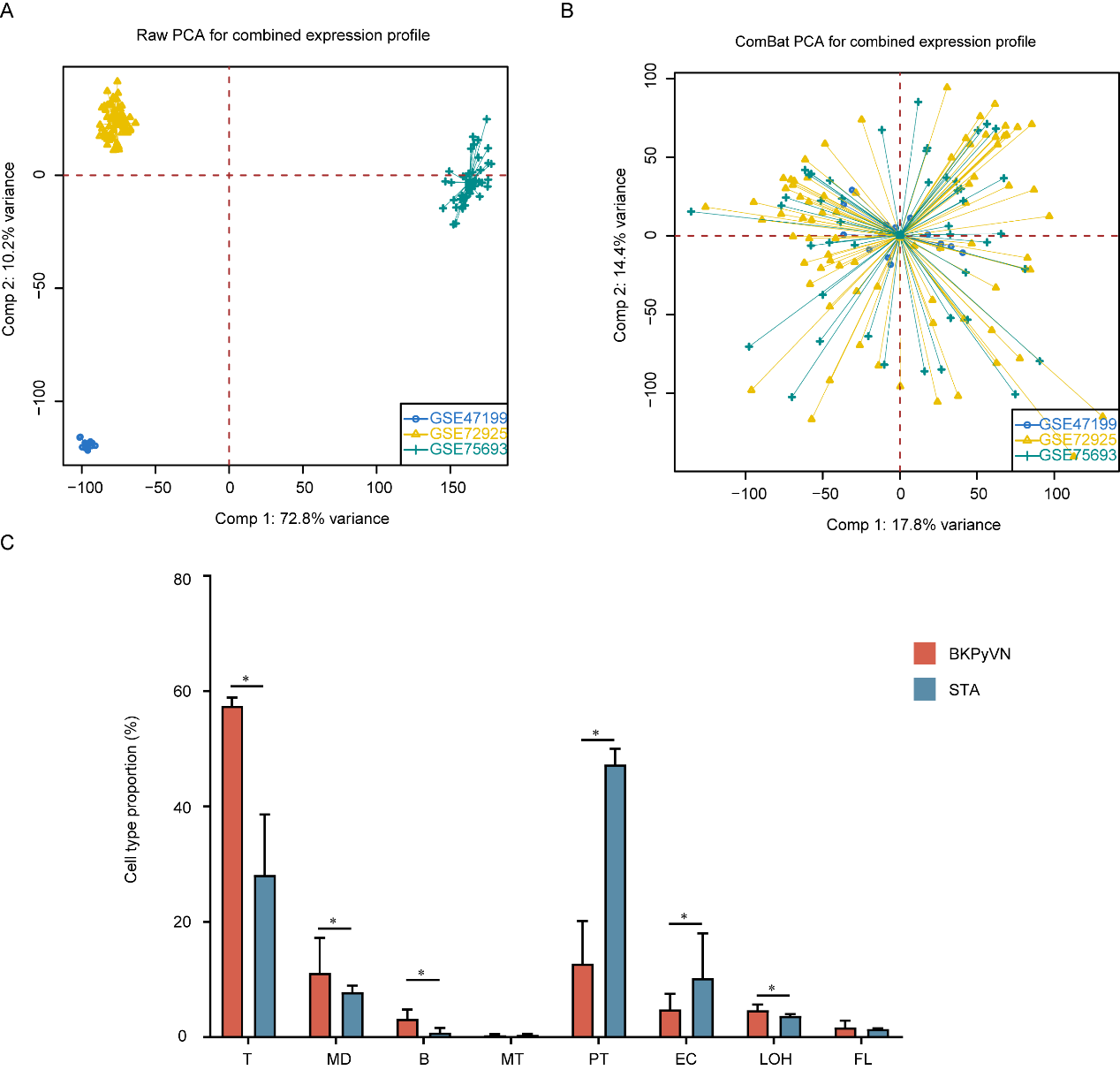


**Fig. S3 Estimation of cell type proportions from bulk RNA-seq of BKPyVN by MuSiC.** (A) Before reducing batch effects, the differences between different groups. (B) The ComBat method was used to reduce batch effects due to non-biological technological biases. (C) Bulk tissue cell type estimation by MuSiC. Statistical comparisons between BKPyVN (n = 28) and STA (n = 109) were conducted using the Wilcoxon rank-sum test, a nonparametric method for two-sample analysis. Data were mean ± SD, * represented statistically significant differences (*p* < 0.05). The red indicated BKPyVN, BK polyomavirus nephropathy; The blue represented STA, stable graft; PCA, principal component analysis.


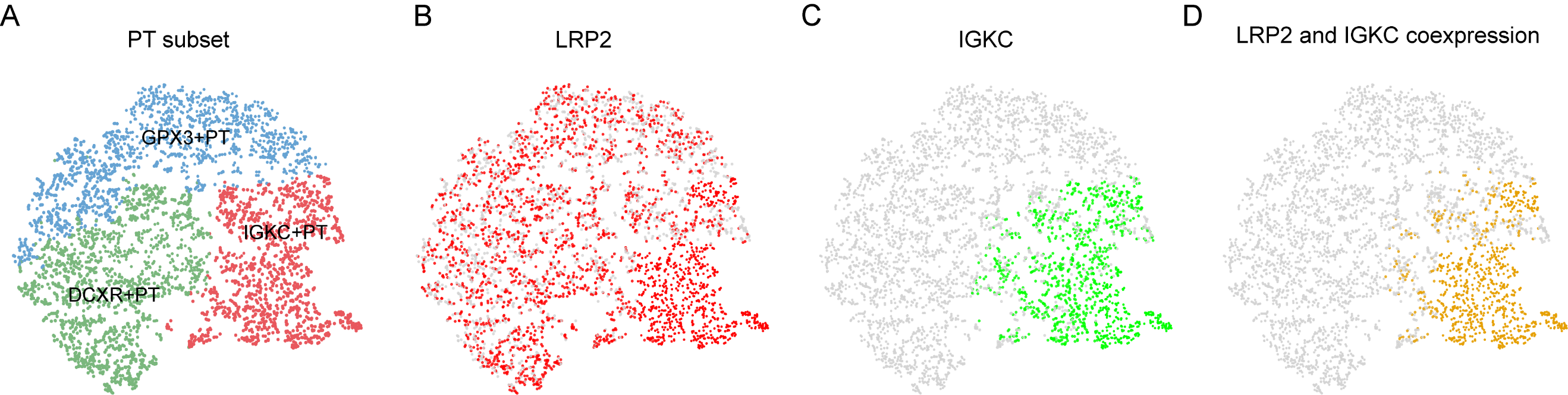


**Fig. S4 The expression of LRP2 and IGKC in the PT subgroup.** (A) Distinct subpopulations within PT cells identified. (B) PT cells expressed LRP2 (red). (C) PT cells demonstrated IGKC expression (green). (D) The LRP2 and IGKC were co-localized in individual PT cells.


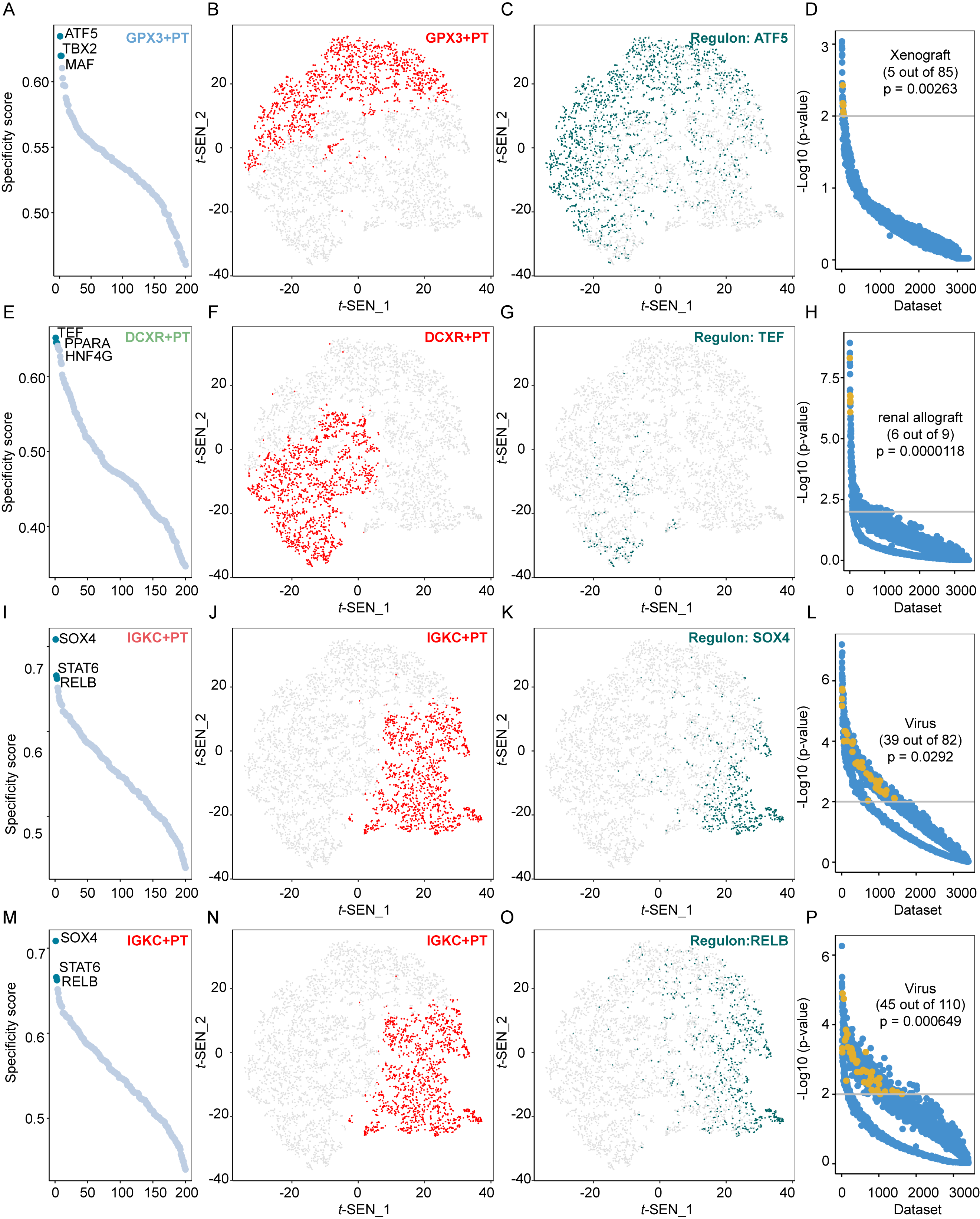


**Figure S5 PT-subsets-specific regulons activity analysis.** (A) Rank for regulons in PT subpopulations based on regulon specificity score (RSS). (B) GPX3+PT subsets were highlighted in the t-SNE map (red dots). (C) Binarized regulon activity scores (RAS) for top regulon on t-SNE map (dark green dots). (D) SEEK co-expression result for target genes of top regulon in different datasets. The X axis represents different datasets, and the Y axis represents the co-expression significance of target genes in each dataset. Term related datasets with significant correlation (P < 0.05) were highlighted by yellow dots. (E-H) Same as (A-D) but for DCXR+PT subcluster. (I-L) Same as (A-D) but for IGKC+PT subsets. (M-P) Same as (A-D) but for IGKC+PT subpopulations with RELB. PT: proximal tubule cells.


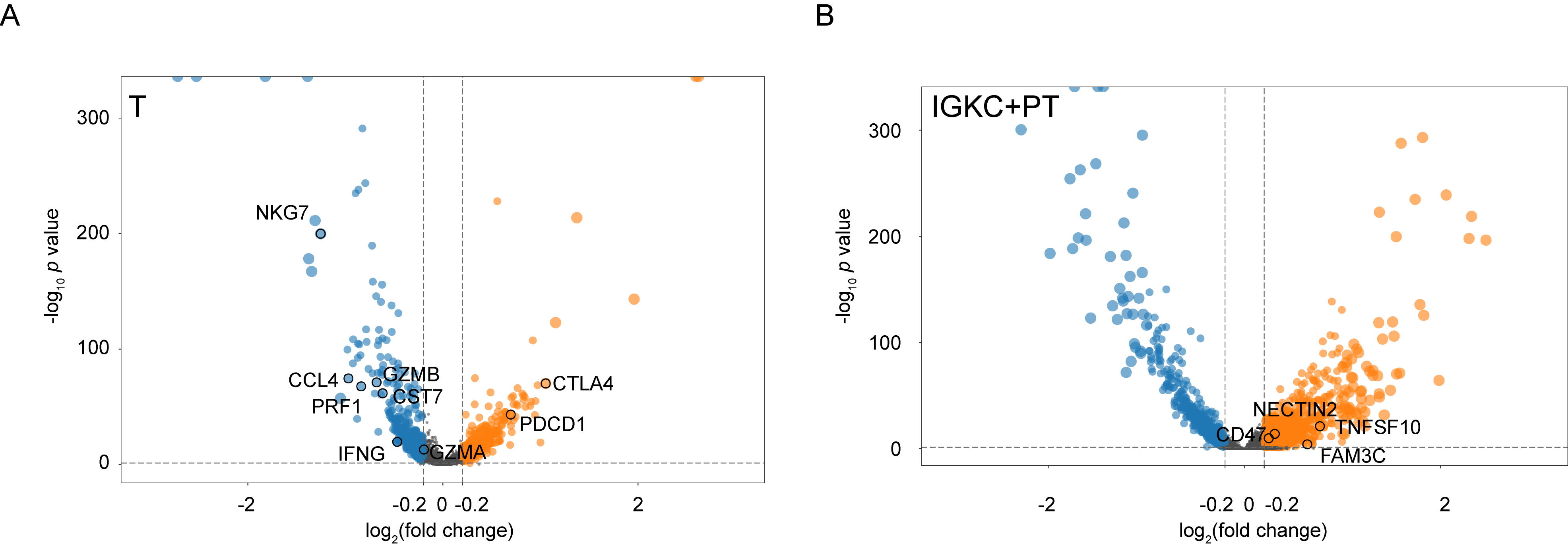


**Fig. S6 Volcano plots of depicting inferred cell-cell interactions between T cells (A) and IGKC+PT subset (B).** The bule dots indicated significant down-regulated genes and the orange dots presented significant up-regulated genes. The cutoff of significant genes was |log2 (fold change)| >0.2 and FDR-adjusted P value < 0.05. The FDR-adjusted *p* value was calculated by the Wilcoxon rank-sum test with FDR correction. In T cells, we annotated genes associated with T cell exhaustion (e.g., PDCD1 and CTLA4) and cytotoxicity-related factors (e.g., NKG7, CCL4, GZMB, PRF1, CST7, IFNG, and GZMA). For IGKC+PT cells, we identified ligands involved in T cell exhaustion-related ligand-receptor pairs, such as CD47, NECTIN2, TNFSF10, and FAM3C.


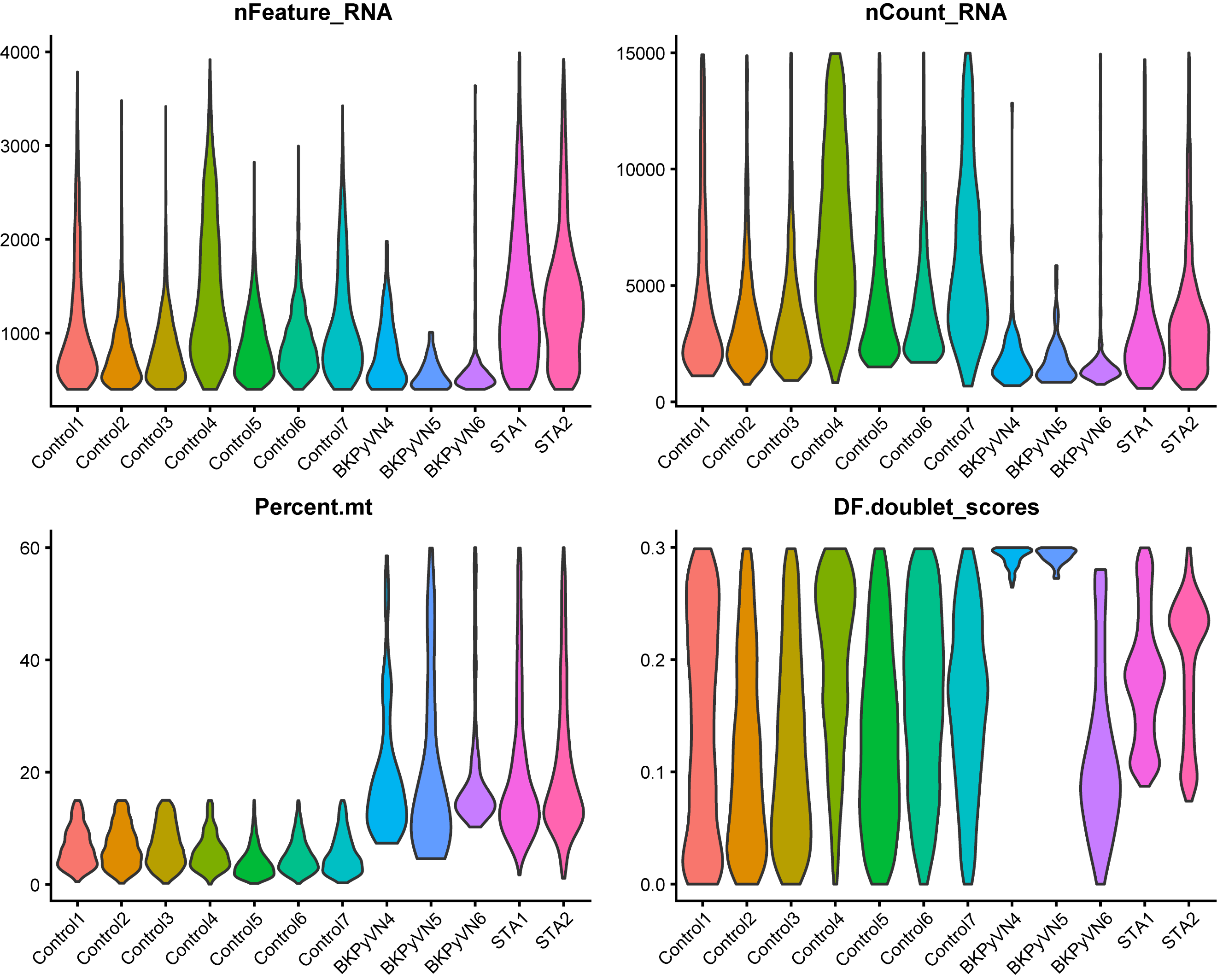


**Fig. S7 After quality control, the number of genes, UMIs, percentage of mitochondrial genes, and doublet scores in each sample.**


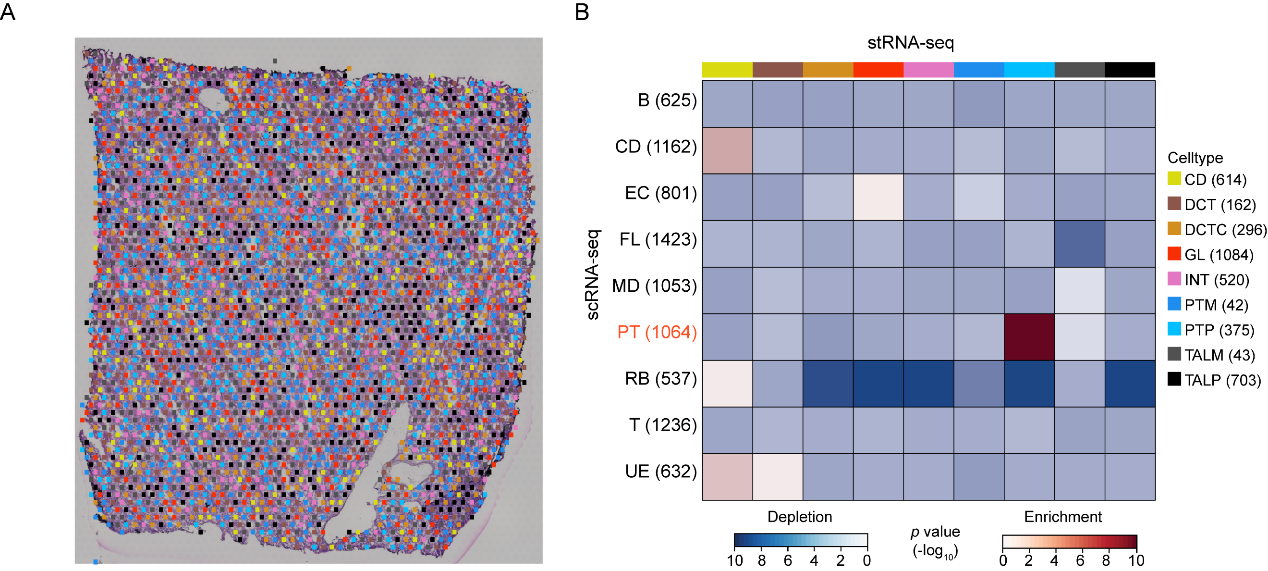


**Fig. S8 Mapping of PT cells from scRNA-seq to stRNA-seq.** (A) The annotation of cell types in stRNA-seq of kidney. (B) The multimodal intersection analysis was used to infer the PT of scRNA-seq location.

**References**

1. Suo S, Zhu Q, Saadatpour A, Fei L, Guo G, Yuan GC. Revealing the Critical Regulators of Cell Identity in the Mouse Cell Atlas. *Cell Rep*. Nov 6 2018;25(6):1436-1445 e3. doi:10.1016/j.celrep.2018.10.045

2. Melo Ferreira R, Sabo AR, Winfree S, et al. Integration of spatial and single-cell transcriptomics localizes epithelial cell-immune cross-talk in kidney injury. *JCI Insight*. Jun 22 2021;6(12)doi:10.1172/jci.insight.147703

3. Moncada R, Barkley D, Wagner F, et al. Integrating microarray-based spatial transcriptomics and single-cell RNA-seq reveals tissue architecture in pancreatic ductal adenocarcinomas. *Nat Biotechnol*. Mar 2020;38(3):333-342. doi:10.1038/s41587-019-0392-8
